# A New Model for the Dynamics of Hepatitis C Infection: Derivation, Analysis and Implications

**DOI:** 10.3390/v10040195

**Published:** 2018-04-13

**Authors:** Philip J. Aston

**Affiliations:** Department of Mathematics, University of Surrey, Guildford, Surrey GU2 7XH, UK; P.Aston@surrey.ac.uk

**Keywords:** HCV infection, mathematical model, steady state solutions, bifurcations

## Abstract

We review various existing models of hepatitis C virus (HCV) infection and show that there are inconsistencies between the models and known behaviour of the infection. A new model for HCV infection is proposed, based on various dynamical processes that occur during the infection that are described in the literature. This new model is analysed, and three steady state branches of solutions are found when there is no stem cell generation of hepatocytes. Unusually, the branch of infected solutions that connects the uninfected branch and the pure infection branch can be found analytically and always includes a limit point, subject to a few conditions on the parameters. When the action of stem cells is included, the bifurcation between the pure infection and infected branches unfolds, leaving a single branch of infected solutions. It is shown that this model can generate various viral load profiles that have been described in the literature, which is confirmed by fitting the model to four viral load datasets. Suggestions for possible changes in treatment are made based on the model.

## 1. Introduction

Viral diseases are major causes of human morbidity and mortality worldwide. Hepatitis C virus (HCV) infection is one of the major contributors in this regard. The WHO recently published a Global Hepatitis Report [1], which provides global estimates regarding various aspects of HCV infection. They report that in 2015:

- Globally, an estimated 71 million people were living with chronic HCV infection;
- An estimated 1.75 million new HCV infections occurred worldwide, while 399,000 people died from end-stage HCV infection and 843,000 were cured;
- 20% of HCV-infected persons (14 million) have been diagnosed, and of these, 7.4% (1.1 million) had started treatment;
- HCV infection affects all regions with the highest reported prevalence in the Eastern Mediterranean and European Regions.

The report also noted that incidence of HCV infection in the USA doubled between 2010 and 2014. Thus, HCV infection is still an issue of major importance for global public health.

In May 2016, the World Health Assembly adopted the Global Health Sector Strategy, which committed to a 65% reduction in mortality and a 90% reduction in incidence by 2030 [1]. Clearly, given the above statistics, there is much work to do in order to achieve these targets. A good understanding of the disease mechanism is vital in designing new drugs for treatment of the infection. This understanding is somewhat hampered by the fact that data can only be easily collected for the viral load, and so, other quantities, such as the concentration of healthy and infected hepatocytes, cannot be determined from patient data.

Mathematical modelling allows the inclusion of all the relevant variables and can help to give insight into disease progression and the effect of treatment. Many different mathematical models have been proposed for HCV infection (as well as for many other viral infections), and we add our own new model to this collection. One of the early HCV models by Neumann et al. [2] was adapted from similar models of HBV and HIV infections. Other models have since been proposed, many of which are variations on this original model. However, these models are not consistent with some of the known facts about HCV infection. Thus, we review some of the information about HCV infection that is available in the biological literature and propose a new model that is consistent with this information.

Having derived a new model, we then analyse the steady state solutions and, unusually for a nonlinear model, we are able to give an analytic solution for the infected steady state solutions in a special case. There are many different viral load profiles described in the literature, and we describe how each of these can be achieved with our model as well as fitting the model to four different viral load profiles. We also make some suggestions for changes in treatment of HCV infection, based on our model predictions.

## 2. A Review of Existing Models of HCV Infection

A number of different mathematical models has been proposed for modelling the dynamics of HCV infection. We first review some of the existing models, before proposing one of our own.

### 2.1. The Neumann Model

Neumann et al. [2] adapted models of HIV and HBV infections to HCV and derived the equations:

(1) $$\begin{array}{ccc} \dot{T} & = & s-dT-(1-\eta )\beta VT \end{array}$$

(2) $$\begin{array}{ccc} \dot{I} & = & (1-\eta )\beta VT-\delta I \end{array}$$

(3) $$\begin{array}{ccc} \dot{V} & = & (1-\epsilon )pI-cV \end{array}$$

where *T* is the concentration of healthy hepatocytes, *I* is the concentration of infected hepatocytes, *V* is the concentration of virions and the dot represents the derivative with respect to time. The treatment parameters η and ϵ correspond to a reduction in the rate of production of infected cells and virions, respectively.

These equations have an uninfected steady state:

(4) $$T_{u}=\frac{s}{d},\;\;\;I_{u}=V_{u}=0$$

which exists for all η and ϵ, together with an infected steady state:

$$\begin{array}{ccc} T_{i} & = & \frac{\delta c}{\beta p(1-\eta )(1-\epsilon )}\\ I_{i} & = & \frac{s}{\delta }-\frac{cd}{\beta p(1-\eta )(1-\epsilon )}\\ V_{i} & = & \frac{sp(1-\epsilon )}{\delta c}-\frac{d}{\beta (1-\eta )} \end{array}$$

The two branches intersect at a bifurcation point at:

(5) $$\left(1-\eta \right)\left(1-\epsilon \right)=\frac{\delta cd}{\beta sp}$$

### 2.2. The First Dahari Model

Dahari et al. [3] analysed the Neumann model and showed that the solutions could exhibit biphasic decline of the viral load under treatment, with an initial rapid decline followed by a slower decline, but that it could not show triphasic behaviour of the viral load, where a plateau exists between the rapid and slow declines. They therefore developed an extended model by adapting the Neumann model, including extra terms for the replication of the healthy and infected hepatocytes. Their model equations are given by:

(6) $$\begin{array}{ccc} \dot{T} & = & s-dT+rT\left(1-\frac{T+I}{T_{\max}}\right)-\left(1-\eta \right)\beta VT \end{array}$$

(7) $$\begin{array}{ccc} \dot{I} & = & \left(1-\eta \right)\beta VT+rI\left(1-\frac{T+I}{T_{\max}}\right)-\delta I \end{array}$$

(8) $$\begin{array}{ccc} \dot{V} & = & (1-\epsilon )pI-cV \end{array}$$

These equations also have an uninfected steady state with $I_{u}=V_{u}=0$ and $T_{u}$ found as the positive solution of the quadratic equation:

(9) $$rT^{2}-T_{\max}\left(r-d\right)T-sT_{\max}=0$$

We note that this quadratic equation has one positive and one negative solution, and clearly, the positive solution is the one of interest. Substituting $T=T_{\max}+\delta T$ into (9) gives the quadratic equation:

(10) $$r\delta T^{2}+T_{\max}\left(r+d\right)\delta T+T_{\max}\left(T_{\max}d-s\right)=0$$

The number of positive or negative solutions of this equation depends on the sign of $T_{\max}d-s$. If this term is positive, then both the solutions are negative, and so, the two solutions of (9) will both lie below $T_{\max}$. However, if this term is negative, then (10) will have one positive and one negative solution. Clearly, the negative solution will correspond to the negative solution of (9), and so, the positive solution must correspond to $T>T_{\max}$. Now, $T_{\max}$ is supposed to be the maximum value for the healthy hepatocytes, and so, we maintain this upper bound provided that the condition:

$$T_{\max}>\frac{s}{d}$$

is satisfied.

Equations (6)–(8) also have an infected steady state given by:

$$\begin{array}{ccc} I_{i} & = & \frac{c(T_{\max}\left(s+\left(r-d\right)T_{i}\right)-rT_{i}^{2})}{T_{i}\left(rc+\left(1-\eta \right)\left(1-\epsilon \right)p\beta T_{\max}\right)}\\ V_{i} & = & \frac{\epsilon p(T_{\max}\left(s+\left(r-d\right)T_{i}\right)-rT_{i}^{2})}{T_{i}\left(rc+\left(1-\eta \right)\left(1-\epsilon \right)p\beta T_{\max}\right)} \end{array}$$

where $T_{i}$ is the positive solution of the quadratic equation:

(11) $${\left(1-\eta \right)}^{2}{\left(1-\epsilon \right)}^{2}\beta^{2}p^{2}T_{\max}T^{2}+c\left(\left(1-\eta \right)\left(1-\epsilon \right)\beta pT_{\max}\left(r-\delta \right)-cr\left(\delta -d\right)\right)T-rsc^{2}=0$$

which also has one positive and one negative solution. Clearly, the positive solution is the only solution of interest. The two branches again intersect at a bifurcation point, which occurs at:

$$\left(1-\eta \right)\left(1-\epsilon \right)=\frac{c(rT_{i}-T_{\max}\left(r-\delta \right))}{p\beta T_{\max}T_{i}}$$

Note, however, that $T_{i}$ also depends on η and ϵ.

### 2.3. The Second Dahari Model

If ribavirin is included as part of the treatment, then it causes some of the virions to be non-infectious. Dahari et al. [4] modelled this by separating the virions into two groups, namely infectious ($V_{I}$) and non-infectious ($V_{NI}$). The equations that they derived are given by:

(12) $$\begin{array}{ccc} \dot{T} & = & s-dT+r_{T}T\left(1-\frac{T+I}{T_{\max}}\right)-\left(1-\eta \right)\beta V_{I}T \end{array}$$

(13) $$\begin{array}{ccc} \dot{I} & = & \left(1-\eta \right)\beta V_{I}T+r_{I}I\left(1-\frac{T+I}{T_{\max}}\right)-\delta I \end{array}$$

(14) $$\begin{array}{ccc} {\dot{V}}_{I} & = & \left(1-\rho \left(t\right)\right)\left(1-\epsilon \right)pI-cV_{I} \end{array}$$

(15) $$\begin{array}{ccc} {\dot{V}}_{NI} & = & \rho \left(t\right)\left(1-\epsilon \right)pI-cV_{NI} \end{array}$$

where ρ(t) is the fraction of virions that are rendered non-infectious due to the effect of ribavirin. Snoeck et al. [5] simplified these equations by taking $r_{T}=r_{I}$ and by making ρ a constant rather than a time-dependent function. We now review the solutions of these simplified equations.

The first three Equations (12)–(14) involve only the three variables *T*, *I* and $V_{I}$, and so, these equations decouple from the fourth. These equations are essentially the same as Equations (6)–(8), except that *V* is replaced by $V_{I}$ and *p* is replaced by (1−ρ)p. Thus, the uninfected steady state is the same as for the first Dahari equations, together with $V_{NI}=0$. The infected steady state is readily obtained from the first Dahari steady state by replacing *p* with (1−ρ)p in the above equations together with:

$$V_{NI,i}=\frac{\rho (1-\epsilon )p}{c}I_{i}$$

Similarly, the bifurcation point at which the two branches intersect occurs at:

$$\left(1-\eta \right)\left(1-\epsilon \right)\left(1-\rho \right)=\frac{c(rT_{i}-T_{\max}\left(r-\delta \right))}{p\beta T_{\max}T_{i}}$$

where $T_{i}$ is the positive solution of (11).

### 2.4. Other Models

There are also a number of variants of the above models in the literature. Herrman et al. [6] used Equations (2) and (3) from the Neumann model, but used a different equation for the healthy hepatocytes, given by:

$$\dot{T}=\gamma \left(T\left(0\right)+I\left(0\right)-T-I\right)$$

We note that this equation does not include the term −βVT associated with the virus infecting the healthy cells. Furthermore, the rate of production of the healthy cells depends on the difference between the maximum value T(0)+I(0) and the total hepatocyte population T+I. After an initial time period, they change δ in (2) to Mδ for some M>1 to represent an inflated loss of infected cells after an initial delay.

Song and Neumann [7] also use Equations (2) and (3) from the Neumann model and supplement this with the equation for *T* given by:

$$\dot{T}=s-dT+aT\left(1-T/T_{\max}\right)-\beta VT$$

In this case, the proliferation is modelled by a logistic function, but the reason for this choice is not given. They also adapt this model by including a saturation term for the generation of infected cells where they replace the βVT term by βVT/(1+αV).

An earlier model by Dahari et al. [8] is very similar to the first Dahari model described above, but with a time-dependent component for the death rate of infected cells and a time-dependent treatment parameter ϵ(t), as well as an extra equation for the dynamics of ALT (alanine aminotransferase). There is also a term q(t)I, which is added to the *T* equation and subtracted from the *I* equation, which represents non-cytolytic clearance of the virus from infected cells. These extra terms were dropped in the later models by Dahari et al. This early model was analysed in more detail by Reluga et al. [9], but with *q* constant, which is then described as a spontaneous cure term.

## 3. A New Model of HCV Infection

Clearly, there are many mathematical models of HCV infection, which are generally similar in nature, but with a variety of different terms occurring in the equations, which represent different biological processes. We now add to this collection of models by developing our own model. We consider the available literature concerning the various dynamical processes that occur during HCV infection and treatment and include those that we consider to be the most important in the model.

### 3.1. Cell Regeneration

The liver is unique among the organs of the body in that it can regenerate itself if part of the liver mass is removed [10]. We therefore begin by considering the dynamics of healthy hepatocytes in an uninfected liver. All of the models described in Section 2 include the terms:

(16) $$\dot{T}=s-dT$$

The standard interpretation of this equation is that new hepatocytes are formed at a constant rate *s* and that a certain fraction *d* of them die. However, hepatocyte turnover is low in a normal liver with more than 99% in the quiescent phase of the cell cycle. But if part of the liver is removed in a partial hepatectomy, then the liver mass is replaced within seven days by replication of the mature hepatocytes [11]. Thus, if the concentration *T* is measured relative to the original volume of the liver, then we model this cell division process by:

$$\dot{T}=r\left(T_{\max}-T\right)$$

so that the concentration is reduced when part of the liver is removed and the division process stops completely when *T* reaches $T_{\max}$. Including the term for cell death gives the equation:

(17) $$\dot{T}=r\left(T_{\max}-T\right)-dT=rT_{\max}-\left(r+d\right)T$$

where *d* must be small to ensure slow turnover in a healthy liver. This equation has steady state $T_{s}=rT_{\max}/\left(r+d\right)$. We note that this equation is essentially the same as (16) with $s=rT_{\max}$ and *d* replaced by r+d. Thus, this equation seems reasonable, but the interpretation of the parameters requires care.

**Figure 1:**
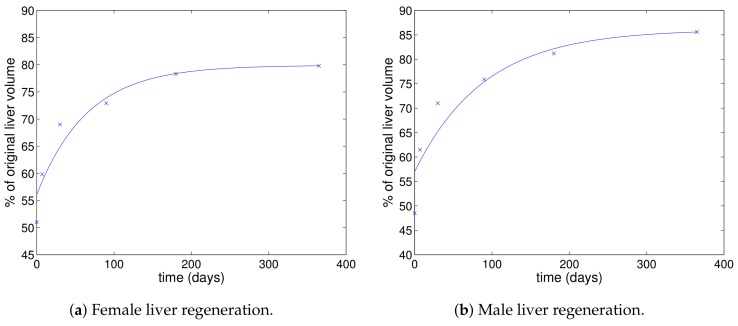
Data for female and male liver regeneration from [12] fitted with a curve of the form $y=a-be^{-ct}$, which is the form of the solution of (17).

It has been found that the liver regenerates itself after surgery with approximately exponential convergence to a steady state [12], as can be seen in Figure 1 (although the regeneration in the first seven days seems to occur more rapidly than predicted by the fitted curve). The solution of Equation (17) also consists of exponential convergence to the steady state. However, care must be exercised in linking these as the plots in Figure 1 are for liver volume, whereas *T* is a concentration of hepatocytes. However, $T/T_{s}$ is the ratio of the reduced number of hepatocytes to the number of hepatocytes in the liver before surgery, which is the same as the ratio of liver volume after surgery to volume before surgery. Dividing (17) by $T_{s}$ gives:

$$\frac{\dot{T}}{T_{s}}=\frac{rT_{\max}}{T_{s}}-\left(r+d\right)\frac{T}{T_{s}}=\left(r+d\right)\left(1-\frac{T}{T_{s}}\right)$$

The solution of this equation is:

$$\frac{T}{T_{s}}=1-Ae^{-(r+d)t}$$

where the constant *A* is determined from the initial condition. This solution can be multiplied by 100 to give a percentage figure, which does not affect the exponential term. Of course, the data shown in Figure 1 indicate that the liver does not completely regain its previous volume, and so, we cannot match this solution precisely to the data. However, we assume that the exponential rate of convergence is the same, which implies that the coefficient of the exponential of the percentage of original liver volume is r+d.

Fitting a function of the form $y=a-be^{-ct}$ to the data shown in Figure 1 gives the curves shown. The corresponding parameter values are a=79.8889, b=23.8424, c=0.0153121 for the female data and a=86.0996, b=29.1096, c=0.0111078 for the male data. Thus, we have $r+d=1.53\times 10^{-2}$ day${}^{-1}$ for the female data and the slightly lower value of $r+d=1.11\times 10^{-2}$ day${}^{-1}$ for the male data.

For an HCV-infected liver, it is assumed in Dahari et al. [8] that the regeneration of the liver occurs through “blind homeostasis” in, which the infected cells regenerate in the same way as the healthy cells. We make this assumption here, but will revisit this issue later. Thus, if we define H=T+I, to be the total concentration of hepatocytes, then the equation for *H* should be the same as our previous Equation (17), but with *T* replaced by *H*, which is therefore given by:

(18) $$\dot{H}=rT_{\max}-\left(r+d\right)H$$

We note that considering only the regeneration terms in the first Dahari model [8] and the variant of the second Dahari model by Snoeck et al. [5], the regeneration terms in the equations for *T* and *I* are:

$$\begin{array}{ccc} \dot{T} & = & rT\left(1-\frac{T+I}{T_{\max}}\right)\\ \dot{I} & = & rI\left(1-\frac{T+I}{T_{\max}}\right) \end{array}$$

Adding these equations gives:

$$\dot{H}=rH\left(1-\frac{H}{T_{\max}}\right)$$

which is a logistic equation for the growth of hepatocytes. The dynamics of the logistic equation ends with exponential convergence to a steady state, but has slower growth at an earlier stage, particularly for initial conditions that are not close to the stable steady state. This does not agree with the above results that show exponential convergence to the steady state, but with more rapid initial growth.

Thus, we allocate the growth of *H*, given by $r(T_{\max}-H)$, to the two pools *T* and *I* in relative proportion to the size of the pools, noting that the size of the pools is not constant over time. We therefore have the equations:

$$\begin{array}{ccc} \dot{T} & = & r\frac{T}{T+I}\left(T_{\max}-\left(T+I\right)\right)\\ \dot{I} & = & r\frac{I}{T+I}\left(T_{\max}-\left(T+I\right)\right) \end{array}$$

Adding these equations gives the regeneration term in (18) as we required.

It is generally assumed that the death rates of the healthy and infected cells are different, as the immune system tries to get rid of the infected hepatocytes, resulting in a higher death rate. Thus, adding in the death terms gives the equations:

$$\begin{array}{ccc} \dot{T} & = & r\frac{T}{T+I}\left(T_{\max}-\left(T+I\right)\right)-dT\\ & = & \frac{rT_{\max}T}{T+I}-\left(r+d\right)T\\ \dot{I} & = & r\frac{I}{T+I}\left(T_{\max}-\left(T+I\right)\right)-\delta I\\ & = & \frac{rT_{\max}I}{T+I}-\left(r+\delta \right)I \end{array}$$

Setting δ=d and adding these equations then gives (18). However, since the death rate for infected cells is higher than for healthy cells, we assume that δ>d.

We made the assumption above that the healthy and infected cells regenerate in the same way, which implies that the total number of hepatocytes *H* is not changed by progression of the infection (except for the increased death rate of the infected cells). However, it is known that “the vast majority of liver diseases are characterized by various levels of damage, loss, and impaired regeneration of mature hepatocytes” [13]. Hepatocyte loss due to disease has been classed as mild (<30%), moderate (30–50%) or severe (>50%) [14], and so, it is clear that infection does result in quite significant loss of hepatocytes. As noted above, the infection also impairs the regeneration of mature hepatocytes, and so, this should be included in the model. We do this by assuming that the regeneration rates of healthy and infected hepatocytes are different (as is done in DebRoy et al. [15]), and so, our equations now become:

$$\begin{array}{ccc} \dot{T} & = & r_{T}\frac{T}{T+I}\left(T_{\max}-\left(T+I\right)\right)-dT\\ & = & \frac{r_{T}T_{\max}T}{T+I}-\left(r_{T}+d\right)T\\ \dot{I} & = & r_{I}\frac{I}{T+I}\left(T_{\max}-\left(T+I\right)\right)-\delta I\\ & = & \frac{r_{I}T_{\max}I}{T+I}-\left(r_{I}+\delta \right)I \end{array}$$

where the two constants $r_{T}$ and $r_{I}$ determine the rate of proliferation of the healthy and infected hepatocytes respectively. DebRoy et al. [15] assume that $r_{I}>r_{T}$ so that the proliferation of infected cells is greater than that for uninfected cells. It has been noted that chronic HCV infection results in increased expression of proliferation markers [11], which suggests an increase in the proliferation rate due to infection. However, it has also been found that progression to the S phase of the cell cycle is blocked, so that there is overall a reduction in the number of cells that complete the cell cycle. This is related to the virus in the cells since “cell cycle progression (is) blocked by individual viral proteins” [11]. We therefore assume that $r_{I}<r_{T}$ so that proliferation of infected cells is reduced. This is also the assumption made by Dahari et al. [4]. A combination of healthy cells becoming infected, a lower rate of regeneration for infected cells and a higher rate of cell death for infected cells is likely to result in a large decrease in the total number of hepatocytes as the infection progresses, as has been observed.

### 3.2. Stem Cells

Thus far, we have considered hepatocyte production only as a result of cell division. However, liver stem cells can also generate hepatocytes, and so, there are two mechanisms for hepatocyte production [10,13,16]. In a healthy liver, which has undergone a partial hepatectomy (where part of the liver has been removed), the healthy hepatocytes self-replicate to restore liver mass, and the contribution of liver stem cells to regeneration “seems to be minimal if any” [10]. However, if there is liver injury, which affects this mechanism of regeneration, such as due to chronic viral hepatitis, then the stem cells become more active as an alternative mechanism of hepatocyte production [10,13,16]. Moreover, “a 50% loss of hepatocytes, together with a significant decrease in proliferation of the remaining mature hepatocytes, is required for an extensive activation of hepatic progenitors” [13]. Thus, it seems that the activation of stem cells is proportional to the degree of infection, with no effect in a healthy liver and an extensive effect in a severely-infected liver. We model this by adding a term sI to the equation for *T*, giving the model equations, which include these two mechanisms of hepatocyte production together with cell death as:

$$\begin{array}{ccc} \dot{T} & = & sI+\frac{r_{T}T_{\max}T}{T+I}-\left(r_{T}+d\right)T\\ \dot{I} & = & \frac{r_{I}T_{\max}I}{T+I}-\left(r_{I}+\delta \right)I \end{array}$$

### 3.3. Infection

Infected hepatocytes are formed when virions enter healthy hepatocytes. The principle of mass action implies that the infection rate is proportional to the product of the concentrations of virions and healthy cells. Adding the infection terms into our earlier equations now gives:

$$\begin{array}{ccc} \dot{T} & = & sI+\frac{r_{T}T_{\max}T}{T+I}-\left(r_{T}+d\right)T-\beta VT\\ \dot{I} & = & \frac{r_{I}T_{\max}I}{T+I}-\left(r_{I}+\delta \right)I+\beta VT\\ \dot{V} & = & -cV-\beta VT \end{array}$$

where we have also included an elimination term in the *V* equation. We note that all of the models discussed above omit the infection term in the *V* equation. In Dahari et al. [4], they point out that if *T* is assumed to be constant, then $-cV-\beta VT=-\left(c+\beta T\right)V=-\tilde{c}V$, and so, the infection term can be considered to be included in the elimination term −cV. However, as the models all include a differential equation for *T*, it is clearly not constant. Moreover, in the case of severe infection, the concentration of healthy hepatocytes may be very low, and so, the assumption that *T* is approximately constant is not valid. Thus, the infection term should also be included in the *V* equation. We note that models for HIV infection are very similar to these models, and in this case, the infection term is also included in all three Equations [17].

### 3.4. The Revised Model

Finally, assuming that infected cells produce virions at a constant rate *p*, we add an extra term into the *V* equation. Treatment with interferon alpha is assumed to both reduce the rate of new infection (η) and to reduce the rate of production of virions (ϵ). Adding in these treatment parameters, we obtain our revised model equations:

(19) $$\begin{array}{ccc} \dot{T} & = & sI+\frac{r_{T}T_{\max}T}{T+I}-\left(r_{T}+d\right)T-\left(1-\eta \right)\beta VT \end{array}$$

(20) $$\begin{array}{ccc} \dot{I} & = & \frac{r_{I}T_{\max}I}{T+I}-\left(r_{I}+\delta \right)I+\left(1-\eta \right)\beta VT \end{array}$$

(21) $$\begin{array}{ccc} \dot{V} & = & (1-\epsilon )pI-cV-(1-\eta )\beta VT \end{array}$$

If the effect of ribavirin is included, which causes some of the virions to be non-infectious, then the model becomes:

(22) $$\begin{array}{ccc} \dot{T} & = & sI+\frac{r_{T}T_{\max}T}{T+I}-\left(r_{T}+d\right)T-\left(1-\eta \right)\beta V_{I}T \end{array}$$

(23) $$\begin{array}{ccc} \dot{I} & = & \frac{r_{I}T_{\max}I}{T+I}-\left(r_{I}+\delta \right)I+\left(1-\eta \right)\beta V_{I}T \end{array}$$

(24) $$\begin{array}{ccc} {\dot{V}}_{I} & = & \left(1-\rho \right)\left(1-\epsilon \right)pI-cV_{I}-\left(1-\eta \right)\beta V_{I}T \end{array}$$

(25) $$\begin{array}{ccc} {\dot{V}}_{NI} & = & \rho \left(1-\epsilon \right)pI-cV_{NI} \end{array}$$

Again, the last equation decouples from the other three, and so, the dynamics of the system is determined by Equations (22)–(24), which are essentially the same as Equations (19)–(21) with $V_{I}$ replaced by *V*. Thus, we only consider Equations (19)–(21) in detail.

### 3.5. Spontaneous Clearance

It has been reported that “about 15–30% of asymptomatic patients and more than 50% of symptomatic patients with acute hepatitis C spontaneously clear the virus during the early phase of infection” [8]. A more recent estimate is that “15–45% of [HCV] infected persons spontaneously clear the virus within 6 months of infection without any treatment” [18]. For infected patients who are cured by treatment, it is also likely that the treatment does not completely eliminate all infected hepatocytes and virions, but that the body is able to clear a small remnant once treatment has stopped. Both of these situations can occur in the equations by ensuring that the uninfected steady state is stable when there is no treatment (ϵ=η=0) for initial conditions sufficiently close to the steady state. However, the Neumann model and the two Dahari models described above do not allow for this possibility as the model equations with the treatment parameters ϵ and η set to zero all have the uninfected steady state as unstable.

The model described in Dahari et al. [8] includes terms (qI) for non-cytolytic clearance of the virus from infected cells. These terms are described in Reluga et al. [9] as being associated with spontaneous cure. In [9], a quasi-steady state approximation is used to reduce the three equations to two. The eigenvalues of the Jacobian evaluated at the uninfected steady state are $\lambda_{1}<0$ and $\lambda_{2}\left(q\right)=\lambda_{2}\left(0\right)-q$, where $\lambda_{2}\left(0\right)$ is the difference of positive terms and so could be positive or negative. Therefore, clearly, the addition of the parameter *q* ensures that a larger region of the parameter space corresponds to spontaneous cure since it moves the eigenvalue $\lambda_{2}$ to the left on the real line. However, this term was dropped in later models used by this group [3,4].

Spontaneous clearance and clearance of infection after cessation of treatment can both be realised in a model by ensuring that the uninfected steady state is stable with no treatment, but with another nearby steady state also existing, which has one unstable and two stable eigenvalues. The two-dimensional stable manifold would then act as a surface that separates out trajectories that converge to the uninfected steady state or move away from this region to a stable infected steady state. Chronic infection would then occur if either the initial conditions at the start of infection lie above this two-dimensional stable manifold, or if, for particular parameter values, the uninfected steady state had become unstable, in which case a complete cure with treatment would also be impossible.

**Figure 2:**
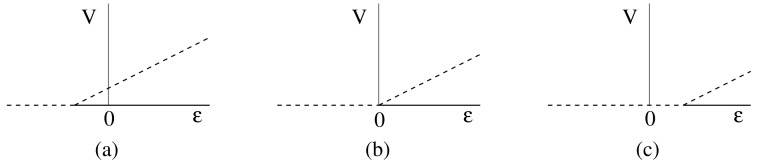
The bifurcation from the uninfected steady state. The bifurcation point occurs for (**a**) ϵ<0, (**b**) ϵ=0, (**c**) ϵ>0. Stable steady states are indicated by solid lines and unstable steady states by dashed lines.

All the models have an uninfected steady state with I=V=0 and T>0. One way in which the scenario just described could occur is if the bifurcation from the uninfected steady state to an infected steady state occurs close to ϵ=0. If the bifurcation point occurs for ϵ<0, at an unphysical parameter value, with the bifurcating branch occurring to the right of the bifurcation point, then there would be two steady state solutions at ϵ=0 with the uninfected steady state being stable and the infected steady state being unstable (see Figure 2a). This situation allows for spontaneous cure or chronic infection, depending on the initial conditions, and for complete cure for a patient once the treatment has reduced the infected hepatocytes and virus levels to a sufficiently low level. However, only a small change in parameter values could move this bifurcation point so that it occurs for a positive value of ϵ (see Figure 2c), which makes the uninfected steady state without treatment unstable. In this case, spontaneous clearance of the infection is not possible, and there will always be relapse of the infection on cessation of treatment, as is observed in some cases [5]. Clearly, only a small change in parameters is required to switch between these cases, which could be due to the variability between patients and would also explain the variability in outcomes for different patients. Various different scenarios will be considered in more detail in Section 8.

### 3.6. Non-Dimensionalisation

Before considering the properties of our new model in detail, we non-dimensionalize it in order to reduce the number of parameters. We non-dimensionalize Equations (19)–(21) by rescaling the variables by the uninfected steady state value of *T*, which is $T_{u}=r_{T}T_{\max}/\left(r_{T}+d\right)$ (note that $T_{u}<T_{\max}$). We therefore define the new non-dimensional variables:

(26) $$x=\frac{(r_{T}+d)T}{r_{T}T_{\max}},\;\;\;y=\frac{(r_{T}+d)I}{r_{T}T_{\max}},\;\;\;z=\frac{(r_{T}+d)V}{r_{T}T_{\max}}$$

together with a new non-dimensional time variable:

$$\tau =(r_{T}+d)t$$

Equations (19)–(21) involve two treatment parameters ϵ and η. Interferon therapy is assumed to partially block viral production (ϵ) and to reduce the rate of production of infected cells (η) [19], which implies that both of the treatment parameters are non-zero. For a bifurcation analysis, it is more convenient to have a single bifurcation parameter, and so, we also make the assumption that:

(27) η=αϵ

for some α>0. It has been shown that the major effect of interferon is to block production or release of virions from an infected cell [2], and this implies that:

(28) α<1

The equations in terms of all these new variables are then given by:

(29) $$\begin{array}{ccc} x^{\prime } & = & Sy+\frac{x}{x+y}-x-\left(1-\alpha \epsilon \right)Bxz \end{array}$$

(30) $$\begin{array}{ccc} y^{\prime } & = & \frac{1}{1+R}\left(\frac{y}{x+y}-y\right)-Dy+\left(1-\alpha \epsilon \right)Bxz \end{array}$$

(31) $$\begin{array}{ccc} z^{\prime } & = & (1-\epsilon )Py-Cz-(1-\alpha \epsilon )Bxz \end{array}$$

where the prime denotes derivatives with respect to τ, and the non-dimensional parameters are given by:

(32) $$S=\frac{s}{r_{T}+d},\;\;\;B=\frac{\beta r_{T}T_{\max}}{{\left(r_{T}+d\right)}^{2}},\;\;\;R=\frac{r_{T}}{r_{I}}-1,\;\;\;D=\frac{r_{T}\delta -r_{I}d}{r_{T}\left(r_{T}+d\right)},\;\;\;P=\frac{p}{r_{T}+d},\;\;\;C=\frac{c}{r_{T}+d}$$

We note that our previous assumptions that $r_{I}<r_{T}$ and d<δ imply that D>0. We also explain our choice of the parameter *R*. The coefficient multiplying the first term in (30) is $r_{I}/r_{T}$, and this satisfies $0<r_{I}/r_{T}<1$. We generally define parameters so that they are positive, but in this case, we have two conditions to satisfy. However, we note that these two conditions are equivalent to the single condition $r_{T}/r_{I}>1$, since it is implicitly assumed that the ratio is finite. Thus, we define $R=r_{T}/r_{I}-1$ and note that R→∞ as $r_{I}/r_{T}\to 0$ and R=0 when $r_{I}/r_{T}=1$. Thus, the single condition R>0 ensures that both conditions on the ratio $r_{I}/r_{T}$ are satisfied. We note therefore that all of these non-dimensional parameters must be positive.

We also require that ϵ∈[0,1), which ensures that the treatment factor 1−ϵ is positive, which then also implies that 1−αϵ is positive using (28).

## 4. Validation of the New HCV Model

In order to validate our new HCV model, we need to check some fundamental mathematical and biological properties of the model. From a mathematical perspective, we need to ensure that the equations are well-posed (a solution exists, is unique and depends continuously on the initial conditions). From a biological perspective, we must show that the solutions are non-negative for all time and are bounded.

All of our variables must be non-negative as they are related to physical quantities, and so, we first show that the non-dimensional Equations (29)–(31) have an invariant region within the octant x,y,z≥0. Using polar coordinates defined by x=rcosθ, y=rsinθ, we see that:

$$\frac{x}{x+y}=\frac{\cos\theta }{\cos\theta +\sin\theta },\;\;\;\;\frac{y}{x+y}=\frac{\sin\theta }{\cos\theta +\sin\theta }$$

and so, the quotients that occur in Equations (29) and (30) are well defined in the limit r→0 along rays with fixed θ for 0≤θ≤π/2. However, this also implies that the vector field is not uniquely defined along the *z* axis, and so, we exclude a neighbourhood of this axis. In a modelling context, the *z* axis (x=y=0) corresponds to there being no healthy or infected hepatocytes, which is unrealistic.

**Theorem** **1.** We define the octant $O=\{\left(x,y,z\right)\in R^{3}:x,y,z\geq 0\}$ and a cylinder around the z axis $C=\{\left(x,y,z\right)\in R^{3}:x^{2}+y^{2}<r_{0}^{2},z\geq 0\}$. The region R=O\C is invariant under the flow of Equations (29)–(31) for sufficiently small $r_{0}>0$. Moreover, the line y=z=0, $x\geq r_{0}$ is an invariant line, corresponding to the dynamics of a healthy liver, on which x converges exponentially to the steady state x=1 for all $x\left(0\right)\geq r_{0}$.

**Proof.**  We first consider the line given by y=z=0, $x\geq r_{0}$. On this line, we have $y^{\prime }=z^{\prime }=0$, and so, it is invariant for all time; and the flow is given by:

$$x^{\prime }=1-x$$

The solution of this equation consists of exponential convergence to the steady state x=1 for all $x\left(0\right)\geq r_{0}$.

The region R is bounded by the planes $P_{1}$ (x=0, $y\geq r_{0}$, z≥0), $P_{2}$ ($x\geq r_{0}$, y=0, z≥0) and $P_{3}$ ($x^{2}+y^{2}\geq r_{0}^{2}$, z=0) together with the surface $S=\{\left(x,y,z\right)\in R^{3}:x^{2}+y^{2}=r_{0}^{2}\}\cap O$. We will show that the vector field given by Equations (29)–(31) on these surfaces is directed inside the region R except for the invariant line.

Taking the inner product of the vector field with the inward pointing normal on the plane $P_{1}$, we see from (29) that:

$$x^{\prime }{|}_{P_{1}}=Sy$$

and so, $x^{\prime }{|}_{P_{1}}>0$ on this plane since $y\geq r_{0}>0$. Thus, the vector field is directed inside R on this plane.

Similarly, from (30), we have:

$$y^{\prime }{|}_{P_{2}}=\left(1-\alpha \epsilon \right)Bxz$$

and so, $y^{\prime }{|}_{P_{2}}>0$ for z>0 (since 1−αϵ>0 as α,ϵ<1) and $y^{\prime }{|}_{P_{2}}=0$ for z=0, which is the invariant line. Thus, the vector field is directed inside R on this plane, except for the invariant line.

From (31), we also have:

$$z^{\prime }{|}_{P_{3}}=\left(1-\epsilon \right)Py$$

and so, $z^{\prime }{|}_{P_{3}}$ is positive everywhere except on the invariant line, since 1−ϵ>0 (as ϵ<1).

Finally, we consider the surface S. The normal to this surface points in the *r* direction when x=rcosθ, y=rsinθ. Differentiating the equation $x^{2}+y^{2}=r^{2}$ with respect to τ and using (29), (30) gives:

$$r^{\prime }=\frac{1+R\cos^{2}\theta }{\left(1+R)(\cos\theta +\sin\theta \right)}+O\left(r\right)$$

Therefore, $r^{\prime }{|}_{r=0}>0$, since θ is in the first quadrant, and this implies that $r^{\prime }{|}_{r=r_{0}}>0$ for sufficiently small $r_{0}$, 0≤θ≤π/2, z≥0. Thus, the vector field points inside R also on this surface.

Thus, we conclude that any trajectory with (x(0),y(0),z(0))∈R=O\C must satisfy (x(t),y(t),z(t))∈R for all t>0, and so, R is invariant. ☐

We now show that our model equations are well-posed on the region R.

**Theorem** **2.** The model Equations (29)–(31) are well-posed on the region R defined in Theorem 1.

**Proof.**  The well-posed condition requires the local existence and uniqueness of solutions of the model equations. For the system of differential equations given by $u^{\prime }=f\left(u,t\right)$, $u\left(t_{0}\right)=u_{0}$ with $u\in R^{n}$, if the function *f* is continuous and satisfies a Lipschitz condition in *u* on a region $|t-t_{0}|\leq \alpha$, $∥u-u_{0}∥\leq \beta$, then it is well-posed [20], and there is then a unique solution on an interval $|t-t_{0}|\leq \delta$ for some δ≤α.

For our model equations given by (29)–(31), the continuity and Lipschitz conditions are satisfied for any such bounded region in the positive octant if the terms x/(x+y) in (29) and y/(x+y) in (30) are excluded. Once the cylinder C in the positive octant is excluded, then these nonlinear terms also satisfy a Lipschitz condition on any bounded region since x+y is bounded below by $r_{0}$. Therefore, our model equations are well-posed. ☐

It remains to show that the solutions are bounded for all time, which we can do provided that a condition on the parameters holds, and this will also imply the global existence of solutions.

**Theorem** **3.** *If:*

(33) min(S,(1−ϵ)P)(1+R)−(1+D(1+R))<0

*then there is a bounded invariant region contained in the region R (defined in Theorem 1). In this case, the solution of Equations (29)–(31) exists and is bounded for all t≥0 for any finite initial conditions.*

We note that this theorem gives a sufficient condition for bounded solutions. If this condition is not satisfied, it may still be the case that the solutions are bounded. We note that the condition (33), when divided by (1+R), requires that the coefficient of the linear decay term in *y* in (30) must exceed one of the coefficients of the linear growth terms in *y* in Equation (29) or (31).

**Figure 3:**
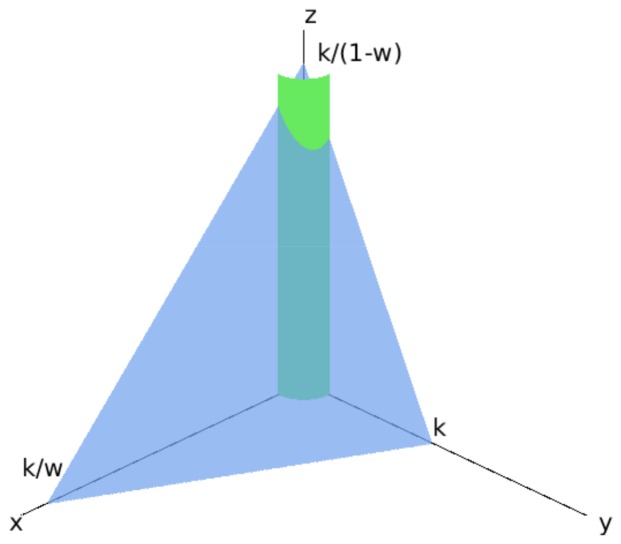
The invariant region in the positive octant bounded by the cylinder C (see Theorem 1) and the plane (34).

**Proof.**  We show that the region R defined in Theorem 1 (the positive octant with a cylinder around the *z* axis removed) together with the additional restriction that:

wx+y+(1−w)z≤k

is a bounded invariant region for k>0 sufficiently large and for all w∈(0,1) provided that a condition on the parameters is satisfied. We note that this region is not bounded for w=0 and w=1, which is why we require that w∈(0,1). This region is shown in Figure 3.

Since we have already shown that the region R is invariant, it remains to show that the vector field is directed inside the bounded region on the plane:

(34) wx+y+(1−w)z=k

when the condition (33) holds.

Using the model Equations (29)–(31), we see that:

$$\begin{array}{ccc} wx^{\prime }+y^{\prime }+\left(1-w\right)z^{\prime } & = & -wx+\left(wS-\frac{1}{1+R}-D+\left(1-w\right)\left(1-\epsilon \right)P\right)y-\left(1-w\right)Cz\\ & & +\frac{wx}{x+y}+\left(\frac{1}{1+R}\right)\left(\frac{y}{x+y}\right) \end{array}$$

and substituting for *z* using (34) gives:

(35) $$\begin{array}{ccc} wx^{\prime }+y^{\prime }+\left(1-w\right)z^{\prime } & = & \left(C-1\right)wx+\left(wS-\frac{1}{1+R}-D+\left(1-w\right)\left(1-\epsilon \right)P+C\right)y-Ck\\ & & +\frac{wx}{x+y}+\left(\frac{1}{1+R}\right)\left(\frac{y}{x+y}\right) \end{array}$$

We first omit the nonlinear terms and so consider the function:

$$f\left(x,y\right)=\left(C-1\right)wx+\left(wS-\frac{1}{1+R}-D+\left(1-w\right)\left(1-\epsilon \right)P+C\right)y-Ck$$

which is a linear function of *x* and *y*. We first show that this function is negative at the three corner points of the triangle where the plane (34) intersects the positive octant O, ignoring the excluded cylinder C at this stage. The function evaluated at these corner points is given by:

$$\begin{array}{ccc} f(0,0) & = & -Ck\\ f(0,k) & = & \left(wS-\frac{1}{1+R}-D+\left(1-w\right)\left(1-\epsilon \right)P\right)k\\ f(k/w,0) & = & -k \end{array}$$

If we assume that:

(36) $$wS-\frac{1}{1+R}-D+\left(1-w\right)\left(1-\epsilon \right)P<0$$

then f(x,y) is negative at all three of the corner points for all k>0. Since it is a linear function of *x* and *y*, this implies that it must also be negative on the boundary and the interior of the triangular region generated by these three points.

We have so far ignored the two nonlinear terms in (35), which are both positive. We note that:

$$0\leq \frac{x}{x+y}\leq 1\;\;\mathrm{for}\mathrm{all}\;\left(x,y\right)\in R$$

and so:

$$0\leq \frac{wx}{x+y}\leq w\;\;\mathrm{for}\mathrm{all}\;\left(x,y\right)\in R$$

Similarly,

$$0\leq \left(\frac{1}{1+R}\right)\left(\frac{y}{x+y}\right)\leq \left(\frac{1}{1+R}\right)\;\;\mathrm{for}\mathrm{all}\;\left(x,y\right)\in R$$

Thus, these two nonlinear terms are uniformly bounded above. Now, the corner points of the linear function f(x,y) scale with *k*, and so, f(x,y) will scale with *k* for all *x* and *y*. Hence, when adding the bounded nonlinear terms to f(x,y), the resulting function will be negative over the whole of the triangular region for sufficiently large *k*. Hence, the vector field points inside the invariant region over the whole of the plane (34) intersected with the positive octant provided that (36) holds and *k* is chosen sufficiently large, and the bounded region (which excludes the cylinder C) is then invariant. For any given initial conditions, *k* must also be chosen sufficiently large so that the initial conditions are contained within the invariant region. The solution must therefore be bounded for all time.

We next consider the condition (36) in more detail. Suppose that (1−ϵ)P<S. Then, when w∈(0,1), we have that:

wS+(1−w)(1−ϵ)P∈((1−ϵ)P,S)

Thus, if (1−ϵ)P−1/(1+R)−D<0 (w=0), then by the intermediate value theorem, there exists a small w>0 such that (36) holds also. A similar argument holds if (1−ϵ)P>S with the condition S−1/(1+R)−D<0. Thus, the optimum value of wS+(1−w)(1−ϵ)P to choose to be as small as possible is *S* if S<(1−ϵ)P or (1−ϵ)P if S>(1−ϵ)P, which is equivalent to min(S,(1−ϵ)P), and this gives the condition (33) in the statement of the theorem after multiplying through by (1+R).

It is a standard result in ode theory that if the function is continuous and satisfies a Lipschitz condition for all t≥0 and on a given domain, then solutions either exist for all time or blow up in finite time [20]. On the bounded domain described above, Equations (29)–(31) satisfy these conditions, using the same argument as in the proof of Theorem 2, and since, we have shown that the solutions are bounded, if (33) holds, then this implies that the solution exists for all t≥0. ☐

## 5. Analysis of the New HCV Model

We now consider the steady states and their bifurcations of the new HCV model given by Equations (19)–(21), together with the dynamical properties of the model.

### 5.1. Assumptions

In our analysis, we make a number of assumptions on the parameters. Rather than having them scattered throughout the text, we now state our main assumptions together for later reference. The key assumptions that we make on the parameters are as follows:

(37) $$\begin{array}{ccc} \alpha & \in & \left(0,1\right) \end{array}$$

(38) $$\begin{array}{ccc} \epsilon & \in & \left[0,1\right) \end{array}$$

(39) $$\begin{array}{ccc} \left(B+C)(B-CR\right) & > & BCP(1+R) \end{array}$$

(40) $$\begin{array}{ccc} D & = & \frac{BP}{B+C} \end{array}$$

The justification for the first two of these conditions was given in Section 3.6, and we assume that these always hold. When we require Assumptions (39) and (40), this will be stated.

Note that if (39) holds, then:

(41) B>CR

must also hold. Moreover, if (39) holds, then:

$$B+C>CP\left(\frac{B(1+R)}{B-CR}\right)$$

We note that B(1+R)>B>B−CR, and so, B(1+R)/(B−CR)>1. It then follows that:

(42) B>C(P−1)

### 5.2. Steady States (S=0)

We first consider the steady states of our revised model (29)–(31) in the special case when S=0. In Section 5.4, we will show how these states are perturbed when S>0.

When S=0, there is a branch of uninfected steady states given by:

(43) $$x_{u}=1,\;\;\;y_{u}=z_{u}=0$$

for all ϵ∈[0,1).

Due to the absence of the *S* term, there is also a state of pure infection given by:

(44) $$x_{p}=0,\;\;\;y_{p}=\frac{1}{1+D(1+R)},\;\;\;z_{p}\left(\epsilon \right)=\frac{(1-\epsilon )P}{C(1+D(1+R))}$$

since, if the only mechanism for cell regeneration is by division and there are no healthy hepatocytes (x=0), then that state will persist as there are no healthy cells to divide. We note that $x_{p}$ and $y_{p}$ are constant, but that $z_{p}$ depends on ϵ.

There is also a branch of infected steady states given by:

$$\begin{array}{ccc} x_{i}\left(\epsilon \right) & = & \frac{C\left(1+D\left(1+R\right)\right)z_{i}\left(\epsilon \right)-\left(1-\epsilon \right)P}{f(z_{i}\left(\epsilon \right),\epsilon )}\\ y_{i}\left(\epsilon \right) & = & \frac{z_{i}\left(\epsilon \right)\left[BCR\left(1-\alpha \epsilon \right)z_{i}\left(\epsilon \right)-\left(B\left(1-\alpha \epsilon \right)+C\right)\right]}{f(z_{i}\left(\epsilon \right),\epsilon )} \end{array}$$

where:

(45) $$f\left(z_{i}\left(\epsilon \right),\epsilon \right)=B\left(1-\alpha \epsilon \right)\left[\left(1-\epsilon \right)PR-1-D\left(1+R\right)\right]z_{i}\left(\epsilon \right)-\left(1-\epsilon \right)P$$

and $z_{i}\left(\epsilon \right)$ is a solution of the quadratic equation:

(46) $$\begin{array}{c} B^{2}CR{\left(1-\alpha \epsilon \right)}^{2}z^{2}+B\left(1-\alpha \epsilon \right)\left(C\left(R+D\left(1+R\right)\right)-B\left(1-\alpha \epsilon \right)\right)z\\ +(1+R)[B(1-\alpha \epsilon )(D-(1-\epsilon )P)+CD]=0 \end{array}$$

These solutions will only be valid provided that $f(z_{i}\left(\epsilon \right),\epsilon )\neq 0$ for all ϵ∈[0,1). We note that f(z,ϵ) is quadratic in ϵ and that for all z>0:

$$\begin{array}{ccc} f(z,1) & = & -B(1-\alpha )[1+D(1+R)]z<0\\ f\left(z,\frac{1}{\alpha }\right) & = & \frac{(1-\alpha )P}{\alpha }>0 \end{array}$$

since α<1 (see (37)). Thus, there exists a function $\tilde{\epsilon }\left(z\right)$ such that $f(z,\tilde{\epsilon }\left(z\right))=0$ with $1<\tilde{\epsilon }\left(z\right)<1/\alpha$ for all z>0, which is clearly outside our range of interest.

The coefficient of $\epsilon^{2}$ in f(z,ϵ) is αBPRz, which is positive for all z>0, and this implies that the second value of ϵ that gives f(z,ϵ)=0 must occur for ϵ<1. In order to avoid the infected steady state branch blowing up in our range of interest, we require that this second root occur for ϵ<0, and this will be the case provided that f(z,0)<0 for all z>0. Now:

f(z,0)=B(PR−1−D(1+R))z−P

The sign of the *z* coefficient in this expression is not clear. However, we assume that (40) holds, and so, substituting for *D* gives:

$$f\left(z,0\right)=-\frac{B(P(B-CR)+B+C)}{B+C}z-P$$

which must be negative for all z>0 using (41). Therefore, assuming that (40) and (41) hold, then f(z,ϵ)≠0 for all ϵ∈[0,1), and so, the infected branch of solutions does not blow up in our region of interest.

### 5.3. Bifurcations (S=0)

We now consider bifurcations that occur in our equations with S=0. In particular, there are bifurcations occurring when the branch of infected steady states intersects the uninfected branch and when it intersects the pure infection branch.

#### 5.3.1. Bifurcation on the Uninfected Branch

**Lemma** **1.** *With S=0, there are two bifurcation points that occur on the uninfected branch of steady state solutions (43) that each give rise to a bifurcating branch of infected steady state solutions. One bifurcation occurs for ϵ>1/α>1, and so is out of our range of interest. The other bifurcation point occurs at $\epsilon =\epsilon_{0}<1$ where*

$$\epsilon_{0}\left\{\begin{array}{cc} <0 & \mathrm{if}\;BP<D(B+C)\\ =0 & \mathrm{if}\;BP=D(B+C)\\ >0 & \mathrm{if}\;BP>D(B+C) \end{array}\right.$$

*The uninfected branch is stable for $\epsilon_{0}<\epsilon <1$ and unstable for $\epsilon <\epsilon_{0}$.*

**Proof.**  The Jacobian matrix J(x,y,z) of Equations (29)–(31) (with S=0) evaluated at the uninfected steady state is given by:

(47) $$J\left(1,0,0\right)=\left[\begin{array}{ccc} -1 & -1 & -(1-\alpha \epsilon )B\\ 0 & -D & (1-\alpha \epsilon )B\\ 0 & (1-\epsilon )P & -C-(1-\alpha \epsilon )B \end{array}\right]$$

Clearly, one eigenvalue of this matrix is −1. We note that the remaining two eigenvalues must be real since, if they were complex, then *y* would oscillate around the steady state value y=0 and similarly for *z*. However, by our invariance result (Theorem 1), this is not possible.

A bifurcation point on the branch of uninfected steady states occurs when detJ(1,0,0)=0, which gives:

(48) $$\alpha BP\epsilon^{2}+\left(\alpha D-\left(1+\alpha \right)P\right)B\epsilon +BP-D\left(B+C\right)=0$$

If ϵ=1+μ, then (48) expressed in terms of μ is given by:

(49) $$\alpha BP\mu^{2}+\left(\alpha D-\left(1-\alpha \right)P\right)B\mu -D\left(B\left(1-\alpha \right)+C\right)=0$$

We note that the constant term is negative (using (37)), and the $\mu^{2}$ coefficient is positive; so the quadratic equation (49) has one positive and one negative solution. This implies that the two solutions of (48) must lie on opposite sides of ϵ=1, and so, one bifurcation point occurs for $\epsilon =\epsilon_{1}>1$ and the other for $\epsilon =\epsilon_{0}<1$.

A similar analysis with ϵ=1/α+μ also shows that the two solutions in μ must have opposite signs, and this implies that $\epsilon_{1}>1/\alpha >1$.

Since $\epsilon_{1}$ is always positive, the sign of the other solution $\epsilon_{0}$ must match the sign of the constant coefficient of (48) since the coefficient of $\epsilon^{2}$ is positive, as stated.

To determine the stability of the uninfected branch, we need to know the signs of the three eigenvalues of J(1,0,0). We have already noted that one eigenvalue is −1, and the remaining two eigenvalues are found from the lower 2×2 block in the Jacobian matrix, which we denote by $J_{1}$. We note that:

$$\mathrm{tr}\left(J_{1}\right)=-C-D-\left(1-\alpha \epsilon \right)B<0$$

since αϵ<1 (using (37), (38)) and:

$$\det\left(J_{1}\right)=-\alpha BP\epsilon^{2}-\left(\alpha D-\left(1+\alpha \right)P\right)B\epsilon -BP+D\left(B+C\right)$$

The determinant is negative for $\epsilon <\epsilon_{0}$ since the coefficient of $\epsilon^{2}$ is negative. A negative determinant implies that the eigenvalues have the opposite sign, and so, there is one positive and one negative eigenvalue, which implies that the uninfected branch is unstable for $\epsilon <\epsilon_{0}$.

At the bifurcation point $\epsilon =\epsilon_{0}$, there is one zero eigenvalue. We note that a double zero eigenvalue is not possible since the other bifurcation point occurs at $\epsilon =\epsilon_{1}>1/\alpha$. Thus, the determinant changes sign at $\epsilon =\epsilon_{0}$ and so is positive for $\epsilon_{0}<\epsilon <1$. A positive determinant and negative trace implies that both of the eigenvalues are negative, and so, the uninfected branch is stable in this range. ☐

Following our discussion regarding spontaneous clearance in Section 3.5, we note that the stability of the uninfected branch for our model agrees with that shown in Figure 2. We initially assume that the bifurcation occurs precisely at ϵ=0 (Figure 2b), as this is the point between the two cases discussed. By Lemma 1, this occurs when BP=D(B+C), and solving this for *D* gives the relation (40).

We now consider the bifurcating branch of infected solutions that arises from the bifurcation point at ϵ=0.

**Lemma** **2.** *When (40) holds, there is a transcritical bifurcation at ϵ=0 on the uninfected branch of solutions. The bifurcating branch of infected solutions is given by:*

$$\begin{array}{ccc} x & = & 1-c\left(BP+B+C\right)\epsilon +O\left(\epsilon^{2}\right)\\ y & = & c\left(B+C\right)\epsilon +O\left(\epsilon^{2}\right)\\ z & = & cP\epsilon +O(\epsilon^{2}) \end{array}$$

*where*

(50) $$c=\frac{\left(1+R)(B+(1+\alpha )C\right)}{\left(B+C)(B-CR)-BCP(1+R\right)}$$

*This branch is unstable for ϵ>0 and stable for ϵ<0.*

**Proof.**  To find the bifurcation equation, we solve (29)+(1+R)(30) and (31) for *x* and *y* in terms of *z* and ϵ, which gives:

(51) $$\begin{array}{ccc} x & = & 1-\left(\frac{BP+B+C}{P}\right)z+zO\left(z,\epsilon \right) \end{array}$$

(52) $$\begin{array}{ccc} y & = & \left(\frac{B+C}{P}\right)z+zO\left(z,\epsilon \right) \end{array}$$

Substituting these into (29) gives the bifurcation equation:

(53) $$c_{1}z\epsilon +c_{2}z^{2}+zO\left({\left(z,\epsilon \right)}^{2}\right)=0$$

where:

$$\begin{array}{ccc} c_{1} & = & -BP^{2}\left(1+R\right)\left(B+\left(1+\alpha \right)C\right)\\ c_{2} & = & BP[(B+C)(B-CR)-BCP(1+R)] \end{array}$$

The two quadratic terms are required for the normal form of a transcritical bifurcation [21]. The trivial solution z=0 of (53) corresponds to the uninfected branch. The non-trivial solution gives a low order solution of the bifurcating infected branch, which is given by:

$$z=-\frac{c_{1}}{c_{2}}\epsilon +O\left(\epsilon^{2}\right)=cP\epsilon +O\left(\epsilon^{2}\right)$$

Substituting this back into (51) and (52) gives the stated expansions for *x* and *y* in terms of ϵ.

With a transcritical bifurcation, the stability of the bifurcating branches is the opposite of the trivial solution, and so, since the uninfected branch is stable for ϵ>0 (see Lemma 1), the bifurcating branch must be unstable for ϵ>0 and stable for ϵ<0. ☐

Clearly, Lemma 2 can be generalised to the case of a bifurcation point occurring at $\epsilon =\epsilon_{0}$ when (40) does not hold.

We note that only one half of the bifurcating branch of infected solutions lies within our invariant region R, and this is the half for which cϵ>0, since then, y,z≥0. In this case, we also have x≤1, as we would expect. Referring back to Figure 2, we would like the valid half of the bifurcating branch to occur for ϵ>0, and this occurs provided that c>0. Clearly the numerator of *c* is positive, and Assumption (39) ensures that the denominator is also positive so that c>0. We then have precisely the situation sketched in Figure 2b.

Now, if condition (40) is perturbed slightly, then the bifurcation point will occur for ϵ either positive or negative. If it occurs for ϵ<0, then the uninfected steady state with no treatment (ϵ=0) is stable, but with a nearby unstable steady state (Figure 2a). Conversely, if the bifurcation point occurs with ϵ>0, then the uninfected steady state with no treatment is unstable (Figure 2c).

#### 5.3.2. Bifurcation on the Pure Infection Branch

In addition to the bifurcation involving the uninfected and infected branches of solutions, there is also a second bifurcation where the infected branch intersects the pure infection branch.

**Lemma** **3.** *With S=0, there are two bifurcation points that occur on the pure infection branch of steady state solutions (44) that each give rise to a bifurcating branch of infected steady state solutions. One bifurcation occurs for ϵ>1/α>1 and so is out of our range of interest. The other bifurcation point occurs at $\epsilon =\epsilon_{2}<1$ where*

$$\epsilon_{2}\left\{\begin{array}{cc} <0 & \mathrm{if}\;BP<CD(1+R)(1+D(1+R))\\ =0 & \mathrm{if}\;BP=CD(1+R)(1+D(1+R))\\ >0 & \mathrm{if}\;BP>CD(1+R)(1+D(1+R)) \end{array}\right.$$

*The pure infection branch is stable for $\epsilon <\epsilon_{2}$ and unstable for $\epsilon_{2}<\epsilon <1$.*

**Proof.**  The bifurcation points can be found by considering the Jacobian matrix derived from Equations (29)–(31) evaluated at the pure infection steady state. This matrix is lower triangular with diagonal entries given by:

(54) $$\begin{array}{ccc} J{\left(x_{p},y_{p},z_{p}\left(\epsilon \right)\right)}_{1,1} & = & \frac{1}{y_{p}}-\left(1-\alpha \epsilon \right)Bz_{p}\left(\epsilon \right)-1\\ J{\left(x_{p},y_{p},z_{p}\left(\epsilon \right)\right)}_{2,2} & = & -\frac{1}{\left(1+R\right)y_{p}}\\ J{\left(x_{p},y_{p},z_{p}\left(\epsilon \right)\right)}_{3,3} & = & -C \end{array}$$

Since the matrix is lower triangular, these diagonal entries are the eigenvalues. Clearly, the second and third eigenvalues are negative. The bifurcation points therefore occur when $J{\left(x_{p},y_{p},z_{p}\left(\epsilon \right)\right)}_{1,1}=0$

Substituting for $y_{p}$ and $z_{p}\left(\epsilon \right)$ from (44) gives the quadratic equation in ϵ:

(55) $$p\left(\epsilon \right)=-BP\alpha \epsilon^{2}+BP\left(1+\alpha \right)\epsilon +CD\left(1+R\right)\left(1+D\left(1+R\right)\right)-BP=0$$

If ϵ=1+μ, then (55) expressed in terms of μ is given by:

$$p\left(1+\mu \right)=-BP\alpha \mu^{2}+BP\left(1-\alpha \right)\mu +CD\left(1+R\right)\left(1+D\left(1+R\right)\right)=0$$

Since the constant term is positive and the $\mu^{2}$ coefficient is negative, this quadratic equation has solutions of opposite sign, which implies that the two solutions of (55) lie on opposite sides of ϵ=1. Clearly, the bifurcation point $\epsilon =\epsilon_{3}>1$ is out of our range of interest, and so, we now consider the other bifurcation point at $\epsilon =\epsilon_{2}<1$.

A similar analysis with ϵ=1/α+μ also shows that the two solutions in μ must have opposite signs, and this implies that $\epsilon_{3}>1/\alpha >1$.

Since $\epsilon_{3}$ is always positive, the sign of $\epsilon_{2}$ will be the opposite of the sign of the constant coefficient in (55), since the $\epsilon^{2}$ coefficient is negative.

The stability of the pure infection steady state is determined by the sign of $J{\left(x_{p},y_{p},z_{p}\left(\epsilon \right)\right)}_{1,1}$ since the other two eigenvalues of $J(x_{p},y_{p},z_{p}\left(\epsilon \right))$ are negative. Combining the two terms in (54) gives a rational function with a positive denominator. The sign is therefore determined by the numerator, which is the expression on the left-hand side of (55). Since $\epsilon_{2}<1<\epsilon_{3}$ and the coefficient of $\epsilon^{2}$ is negative, then clearly, $J{\left(x_{p},y_{p},z_{p}\left(\epsilon \right)\right)}_{1,1}<0$ for $\epsilon <\epsilon_{2}$, and so, the pure infection steady state is stable in this range. There is no possibility of a double root occurring as the two roots lie on opposite sides of ϵ=1, and so, this quadratic must change sign at the bifurcation point. Therefore, the pure infection solution is unstable for $\epsilon_{2}<\epsilon <1$ as claimed. ☐

It is natural to assume that the pure infection state is stable when there is no treatment (ϵ=0), and from Lemma 3, we see that this occurs provided that:

(56) BP−CD(1+R)(1+D(1+R))>0

which implies that $\epsilon_{2}>0$. However, we will show in Section 5.4.4 that this is in fact not required for our later analysis of the model. We also note that with the assumption (40), the inequality (56) becomes:

$$\left(B+C\right)\left(B-CR\right)-BCP{\left(1+R\right)}^{2}>0$$

Clearly, this is very similar to the inequality in (39), but the fact that the last term is squared implies that the sign of this expression cannot be determined using (39) and so could be positive or negative.

We now consider the bifurcating branch of infected solutions that arises from the bifurcation point at $\epsilon =\epsilon_{2}$.

**Lemma** **4.** *The bifurcating branch of infected solutions arising from the transcritical bifurcation on the pure infection branch at $\epsilon =\epsilon_{2}$ is given by:*

(57) $$\begin{array}{ccc} x & = & -\frac{c_{3}}{c_{4}}\left(\epsilon -\epsilon_{2}\right)+O\left({\left(\epsilon -\epsilon_{2}\right)}^{2}\right)\\ y & = & y_{p}+c_{5}\left(\epsilon -\epsilon_{2}\right)+O\left({\left(\epsilon -\epsilon_{2}\right)}^{2}\right)\\ z & = & z_{p}\left(\epsilon_{2}\right)+\left(c_{6}-\frac{P}{C}y_{p}\right)\left(\epsilon -\epsilon_{2}\right)+O\left({\left(\epsilon -\epsilon_{2}\right)}^{2}\right)\\ & = & z_{p}\left(\epsilon \right)+c_{6}\left(\epsilon -\epsilon_{2}\right)+O\left({\left(\epsilon -\epsilon_{2}\right)}^{2}\right) \end{array}$$

*where:*

$$\begin{array}{ccc} c_{3} & = & B\left(\left(1-\alpha \epsilon_{2}\right)\frac{Py_{p}}{C}+\alpha z_{p}\left(\epsilon_{2}\right)\right)\\ c_{4} & = & \left(1-\alpha \epsilon_{2}\right)Bz_{p}\left(\epsilon_{2}\right)\left(\left(1-\alpha \epsilon_{2}\right)B\left(\frac{1}{C}-\left(1+R\right)z_{p}\left(\epsilon_{2}\right)\right)-\frac{R}{y_{p}}\right)\\ c_{5} & = & -\left(\left(1-\alpha \epsilon_{2}\right)B\left(1+R\right)y_{p}z_{p}\left(\epsilon_{2}\right)-1\right)\frac{c_{3}}{c_{4}}\\ c_{6} & = & z_{p}\left(\epsilon_{2}\right)\left(\left(1-\alpha \epsilon_{2}\right)B\left(\frac{1}{C}-\left(1+R\right)z_{p}\left(\epsilon_{2}\right)\right)+\frac{1}{y_{p}}\right)\frac{c_{3}}{c_{4}} \end{array}$$

*This branch is stable for $\epsilon >\epsilon_{2}$ and unstable for $\epsilon <\epsilon_{2}$.*

**Proof.**  Since the pure infection steady state has x=0, we will derive the bifurcation equation in terms of *x* and $\epsilon -\epsilon_{2}$. To do this, we solve Equations (30) and (31) for *y* and *z* in terms of *x* and $\epsilon -\epsilon_{2}$, which gives:

(58) $$\begin{array}{ccc} y & = & y_{p}+\left(\left(1-\alpha \epsilon_{2}\right)B\left(1+R\right)y_{p}z_{p}\left(\epsilon_{2}\right)-1\right)x+xO\left(x,\left(\epsilon -\epsilon_{2}\right)\right)\\ z & = & z_{p}\left(\epsilon_{2}\right)+z_{p}\left(\epsilon_{2}\right)\left(\left(1-\alpha \epsilon_{2}\right)B\left(\left(1+R\right)z_{p}\left(\epsilon_{2}\right)-\frac{1}{C}\right)-\frac{1}{y_{p}}\right)x \end{array}$$

(59) $$\begin{array}{c} -\frac{P}{C}y_{p}\left(\epsilon -\epsilon_{2}\right)+O\left({\left(x,\left(\epsilon -\epsilon_{2}\right)\right)}^{2}\right) \end{array}$$

Substituting these into (29) gives the bifurcation equation:

(60) $$c_{3}x\left(\epsilon -\epsilon_{2}\right)+c_{4}x^{2}+xO\left({\left(x,\left(\epsilon -\epsilon_{2}\right)\right)}^{2}\right)$$

where:

$$\begin{array}{ccc} c_{3} & = & B\left(\left(1-\alpha \epsilon_{2}\right)\frac{Py_{p}}{C}+\alpha z_{p}\left(\epsilon_{2}\right)\right)\\ c_{4} & = & \left(1-\alpha \epsilon_{2}\right)Bz_{p}\left(\epsilon_{2}\right)\left(\left(1-\alpha \epsilon_{2}\right)B\left(\frac{1}{C}-\left(1+R\right)z_{p}\left(\epsilon_{2}\right)\right)-\frac{R}{y_{p}}\right) \end{array}$$

The two quadratic terms are required for the normal form of a transcritical bifurcation [21]. The trivial solution x=0 of (60) corresponds to the pure infection branch. The non-trivial solution gives a low order solution of the bifurcating infected branch, which is given by (57). Substituting for *x* back into (58) and (59) gives the stated expansions for *y* and *z* in terms of $\left(\epsilon -\epsilon_{2}\right)$. We also note from the pure infection steady states (44) that:

$$z_{p}\left(\epsilon_{2}\right)-\frac{P}{C}y_{p}\left(\epsilon -\epsilon_{2}\right)=z_{p}\left(\epsilon \right)$$

and this is used to derive the stated second form of *z*.

The stability of the bifurcating branches at a transcritical bifurcation is the opposite of the trivial solution, and so, since the pure infection branch is stable for $\epsilon <\epsilon_{2}$ (see Lemma 3), the bifurcating branch must be unstable for $\epsilon <\epsilon_{2}$ and stable for $\epsilon >\epsilon_{2}$. ☐

We note that $c_{3}>0$ using (37) and the fact that $\epsilon_{2}<1$ (see Lemma 3). However, $c_{4}$ could be positive or negative depending on the values of the parameters in the model. Since *x* cannot be negative, we see from (57) that:

- if $c_{4}<0$, then x(ϵ) has a positive slope, and valid solutions exist locally only for $\epsilon \geq \epsilon_{2}$;
- if $c_{4}>0$, then x(ϵ) has a negative slope, and valid solutions exist locally only for $\epsilon \leq \epsilon_{2}$.

Furthermore, if $c_{4}>0$, then:

$$\left(1-\alpha \epsilon_{2}\right)B\left(\frac{1}{C}-\left(1+R\right)z_{p}\left(\epsilon_{2}\right)\right)>\frac{R}{y_{p}}$$

and this implies that:

$$c_{6}>\frac{z_{p}\left(\epsilon_{2}\right)\left(1+R\right)c_{3}}{c_{4}y_{p}}$$

and so, $c_{6}>0$ also. However, if $c_{4}<0$, then $c_{6}$ could be positive or negative.

**Figure 4:**
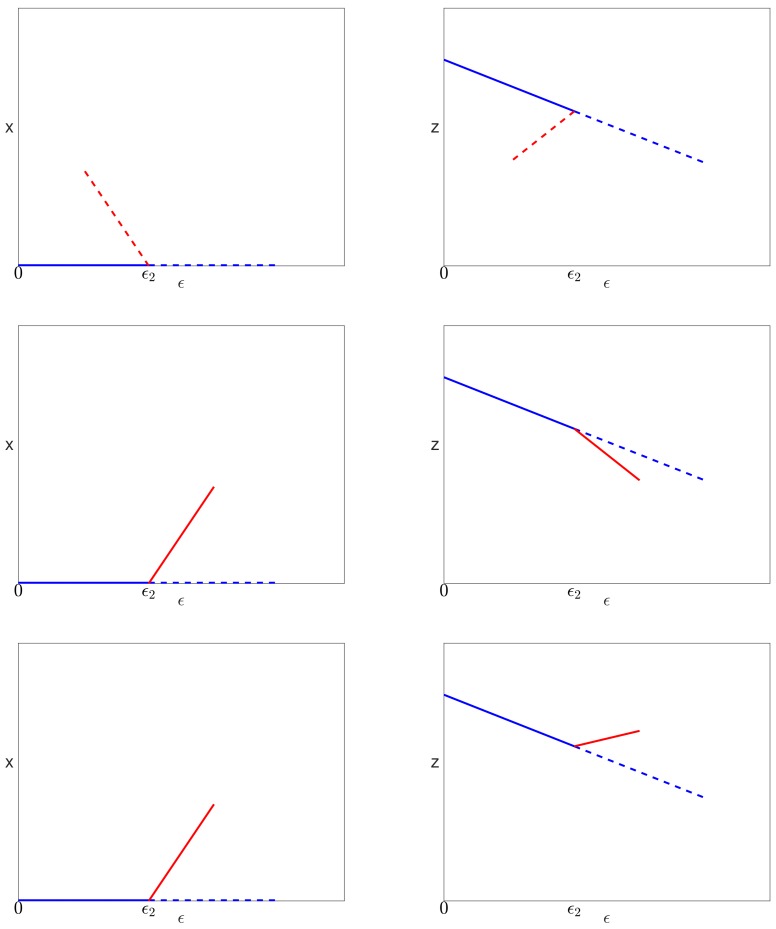
Solutions near the bifurcation involving the infected (red) and pure infection (blue) branches. Solid lines indicate stable solutions, while dashed lines represent unstable solutions. **Top**: $c_{4}>0$. **Middle**: $c_{4}<0$, $c_{6}<0$. **Bottom**: $c_{4}<0$, $c_{6}>0$.

The solutions of Equations (29)–(31) near the bifurcation point are sketched in Figure 4 for the three possible cases associated with different signs of $c_{4}$ and $c_{6}$.

#### 5.3.3. The Infected Steady State Branch (S=0)

We have found that the infected steady state branch intersects both the uninfected and the pure infection branches of steady states at transcritical bifurcation points. We next consider what happens to the infected branch in between these two bifurcations.

**Theorem** **4.** *We assume that (39) and (40) hold and that:*

(61) $$R>\frac{\sqrt{2}-1}{2}$$

*Then, the bifurcation point at ϵ=0 on the uninfected branch of solutions and the bifurcation point at $\epsilon =\epsilon_{2}$ on the pure infection branch of solutions are connected by a continuous branch of infected steady state solutions. There is a single limit point on this branch of infected solutions, which occurs between the two bifurcations when $c_{4}<0$ or after the bifurcation on the pure infection branch when $c_{4}>0$. The limit point occurs at $\epsilon =\epsilon_{4}$ where $\epsilon_{2}<\epsilon_{4}<1/\alpha$. The three possible cases, associated with different signs of the coefficients $c_{4}$ and $c_{6}$, are shown in Figure 4.*

**Remark** **1.** We conjecture that Theorem 4 holds for all R>0, so that condition (61) is unnecessary. However, we have been unable to prove this. Some evidence to support this conjecture is included in the proof. Condition (61) is not overly restrictive though, as it implies that $r_{I}/r_{T}<2/\left(\sqrt{2}+1\right)=0.8284$.

**Proof.**  Substituting for *D* using (40) into (46), we obtain an equation involving *z* and ϵ to be solved for the infected branch of solutions given by:

(62) $$\begin{array}{c} g\left(z,\epsilon \right)=BCR\left(B+C\right){\left(1-\alpha \epsilon \right)}^{2}z^{2}+(BCP\left(1+R\right)+\left(B+C\right)\left(CR-B\left(1-\alpha \epsilon \right)\right)\left(1-\alpha \epsilon \right)z\\ +\epsilon P(1+R)((B+C)(1-\alpha \epsilon )+\alpha C)=0 \end{array}$$

To find limit points on the infected steady state branch [21], we must solve the two equations:

$$g\left(z,\epsilon \right)=g_{z}\left(z,\epsilon \right)=0$$

and check the non-degeneracy conditions:

(63) $$g_{zz}\left(z,\epsilon \right)\neq 0,\;\;\;\;g_{\epsilon }\left(z,\epsilon \right)\neq 0$$

Since g(z,ϵ) is quadratic in *z*, then clearly, $g_{z}\left(z,\epsilon \right)$ is linear in *z*, and the *z* coefficient is strictly positive. Thus, the second equation can be solved for *z* and substituted back into the first equation, which gives the quadratic equation in ϵ:

(64) $$A_{1}\epsilon^{2}+B_{1}\epsilon +C_{1}=0$$

where:

$$\begin{array}{ccc} A_{1} & = & -\alpha B{\left(B+C\right)}^{2}\left(4CPR\left(1+R\right)+\alpha B\right)\\ B_{1} & = & CP(1+R)(2R(B+(1+\alpha )C)-\alpha B)+\alpha (B+C)(B-CR)\\ C_{1} & = & -{\left(\left(B+C\right)\left(B-CR\right)-BCP\left(1+R\right)\right)}^{2} \end{array}$$

The discriminant of the quadratic is given by:

(65) $$\Delta =16BC^{2}PR\left(1+R\right){\left(B+C\right)}^{2}\Delta_{1}\Delta_{2}$$

where:

(66) $$\begin{array}{ccc} \Delta_{1} & = & BP(1+R)+\alpha (B-CR) \end{array}$$

(67) $$\begin{array}{ccc} \Delta_{2} & = & \left(B+C)(\alpha B+R(B+C))-\alpha BCP(1+R\right) \end{array}$$

We have assumed that (39) holds, and this implies (41) also; this gives $\Delta_{1}>0$. We also note that $\Delta_{2}$ can be expressed as:

$$\Delta_{2}=\left({\left(B+C\right)}^{2}-\alpha BCP\right)R+\alpha B\left(B+C-CP\right)$$

Assumption (39) implies that (42) holds, which implies that the second term is positive. Moreover,

$$\begin{array}{ccc} {\left(B+C\right)}^{2}-\alpha BCP & > & (B+C)CP-\alpha BCP\;\;\;\mathrm{using}\;(42)\\ & = & CP(B(1-\alpha )+C)\\ & > & 0 \end{array}$$

using (37), and this implies that $\Delta_{2}>0$ also. Therefore, Δ>0, and so, the quadratic Equation (64) will have two distinct solutions. We therefore expect to have two limit points on the infected solution branch(es). Now, $g_{zz}\left(z,\epsilon \right)=2BCR\left(B+C\right){\left(1-\alpha \epsilon \right)}^{2}>0$ using (37), (38), and so, the first non-degeneracy condition in (63) is satisfied. However, it is not easy to verify the second non-degeneracy condition. We now use a different approach to get further information regarding the infected solution branches, which will also confirm the existence of two limit points.

To get a more complete picture of the solutions, we substitute:

(68) $$z=z_{0}+\frac{z_{1}+\delta z}{{\tilde{\epsilon }}_{1}+\delta \epsilon },\;\;\;\;\epsilon ={\tilde{\epsilon }}_{0}+{\tilde{\epsilon }}_{1}+\delta \epsilon$$

into (62). The parameters $z_{0}$, $z_{1}$, ${\tilde{\epsilon }}_{0}$ and ${\tilde{\epsilon }}_{1}$ can be solved for in terms of the parameters so that our equation reduces to:

(69) $$\frac{h_{1}\delta \epsilon^{2}}{h_{0}}-\frac{h_{2}\delta z^{2}}{h_{0}}=1$$

where:

$$z_{0}=\frac{1}{2CR},\;\;\;\;z_{1}=\frac{BP(1+R)+R(B+C)}{2\alpha BR(B+C)},$$

$${\tilde{\epsilon }}_{0}=\frac{1}{\alpha },\;\;\;\;{\tilde{\epsilon }}_{1}=-\frac{C[\alpha R(B+C)+P(1+R)(2R(B+C(1-\alpha ))+\alpha B)]}{\alpha (B+C)(\alpha B+4CPR(1+R))},$$

$$h_{0}=\frac{P\left(1+R\right)\Delta_{1}\Delta_{2}}{4BRh_{1}},\;\;\;\;h_{1}=\frac{\alpha (B+C)(\alpha B+4CPR(1+R))}{4CR},\;\;\;h_{2}=\alpha^{2}BCR\left(B+C\right)$$

Clearly, $h_{1},h_{2}>0$, and we showed above that $\Delta_{1}$, $\Delta_{2}>0$; so, $h_{0}>0$ also. Thus, (69) is the equation of a hyperbola, and the two solution branches are given in parametric form by:

$$\delta z\left(\beta \right)=\sqrt{\frac{h_{0}}{h_{2}}}\sinh\beta ,\;\;\;\;\delta \epsilon \left(\beta \right)=\pm \sqrt{\frac{h_{0}}{h_{1}}}\cosh\beta$$

These solutions exist for all δz and for $\left|\delta \epsilon \right|\geq \sqrt{h_{0}/h_{1}}$. This gives rise to parametric solutions of (62) given by:

(70) $$z\left(\beta \right)=z_{0}+\frac{z_{1}+\delta z\left(\beta \right)}{{\tilde{\epsilon }}_{1}+\delta \epsilon \left(\beta \right)},\;\;\;\;\epsilon \left(\beta \right)={\tilde{\epsilon }}_{0}+{\tilde{\epsilon }}_{1}+\delta \epsilon \left(\beta \right)$$

Limit points on these branches occur when:

$$\frac{d\epsilon }{dz}=\frac{d\epsilon /d\beta }{dz/d\beta }=0$$

Now, β=0 is the unique solution of the equation dϵ/dβ=0 and:

$${\left.\frac{dz}{d\beta }\right|}_{\beta =0}=\frac{\sqrt{h_{0}/h_{2}}}{{\tilde{\epsilon }}_{1}\pm \sqrt{h_{0}/h_{1}}}$$

For this derivative to be finite, we clearly require ${\tilde{\epsilon }}_{1}\pm \sqrt{h_{0}/h_{1}}\neq 0$. Now, ${\tilde{\epsilon }}_{1}<0$, and so, ${\tilde{\epsilon }}_{1}-\sqrt{h_{0}/h_{1}}<0$; however, it is possible that ${\tilde{\epsilon }}_{1}+\sqrt{h_{0}/h_{1}}=0$. However, we will show later that the right-hand (+) branch is outside our range of interest, and so, it does not matter whether this quantity is zero or non-zero. Thus, on the left-hand (−) branch, we have:

$${\left.\frac{dz}{d\beta }\right|}_{\beta =0}=\frac{\sqrt{h_{0}/h_{2}}}{{\tilde{\epsilon }}_{1}-\sqrt{h_{0}/h_{1}}}\neq 0$$

and so, dϵ/dz=0 when β=0. This point will be a quadratic limit point provided that the non-degeneracy condition $d^{2}\epsilon /dz^{2}{|}_{\beta =0}\neq 0$ is satisfied. It is a matter of calculation to show that for the left-hand branch:

$${\left.\frac{d^{2}\epsilon }{dz^{2}}\right|}_{\beta =0}={\left.\frac{d^{2}\epsilon /d\beta^{2}}{{\left(dz/d\beta \right)}^{2}}\right|}_{\beta =0}=-\sqrt{\frac{h_{2}}{h_{0}h_{1}}}{\left({\tilde{\epsilon }}_{1}-\sqrt{h_{0}/h_{1}}\right)}^{2}\neq 0$$

again using the fact that ${\tilde{\epsilon }}_{1}-\sqrt{h_{0}/h_{1}}<0$, and so, we do indeed have a quadratic limit point when β=0. Thus, the limit point on the left-hand branch of solutions occurs at:

$$z\left(0\right)=z_{0}+\frac{z_{1}}{{\tilde{\epsilon }}_{1}-\sqrt{h_{0}/h_{1}}},\;\;\;\;\epsilon \left(0\right)={\tilde{\epsilon }}_{0}+{\tilde{\epsilon }}_{1}-\sqrt{h_{0}/h_{1}}$$

and two solutions exist for each ϵ<ϵ(0), which is also confirmed by the negative second derivative.

We note that there are no solutions of (69) when δϵ=0, and this corresponds to $\epsilon ={\tilde{\epsilon }}_{0}+{\tilde{\epsilon }}_{1}$. We now show that $\epsilon_{2}<{\tilde{\epsilon }}_{0}+{\tilde{\epsilon }}_{1}<1/\alpha$. The right-hand inequality is clearly satisfied since ${\tilde{\epsilon }}_{0}=1/\alpha$ and ${\tilde{\epsilon }}_{1}<0$. To verify the left-hand inequality, we consider the quadratic equation (55). We have already shown in the proof of Lemma 3 that the two solutions $\epsilon_{2}$ and $\epsilon_{3}$ of this equation satisfy $\epsilon_{2}<1<\epsilon_{3}$. Since the coefficient of $\epsilon^{2}$ is negative, this implies that the quadratic function p(ϵ) is positive if and only if $\epsilon_{2}<\epsilon <\epsilon_{3}$. If $p({\tilde{\epsilon }}_{0}+{\tilde{\epsilon }}_{1})>0$, then this implies that $\epsilon_{2}<{\tilde{\epsilon }}_{0}+{\tilde{\epsilon }}_{1}$ as required. It can be shown that:

(71) $$p\left({\tilde{\epsilon }}_{0}+{\tilde{\epsilon }}_{1}\right)=\frac{BPC(a_{3}P^{3}+a_{2}P^{2}+a_{1}P+a_{0})}{\alpha {\left(B+C\right)}^{2}{\left(\alpha B+4CPR\left(1+R\right)\right)}^{2}}$$

where $a_{0},a_{1},a_{3}>0$, using (37) and (41). The remaining coefficient, $a_{2}$, is given by:

$$a_{2}=C{\left(1+R\right)}^{2}\left(b_{2}B^{2}+b_{1}B+b_{0}\right)$$

where $b_{0},b_{1}>0$ and:

$$b_{2}=\left(8\alpha^{2}-8\alpha +4\right)R^{2}+4\alpha^{2}R-\alpha^{2}$$

Now, $b_{2}$ is negative for α>0 and sufficiently small *R*. Substituting $R=\left(\sqrt{2}-1\right)/2+\tilde{R}$ into $b_{2}$ gives:

$$b_{2}=\left(8\alpha^{2}-8\alpha +4\right){\tilde{R}}^{2}+\frac{4(2\sqrt{2}-1)}{7}\left(7\alpha^{2}+\left(2\sqrt{2}-6\right)\alpha +3-\sqrt{2}\right)\tilde{R}+\left(3-2\sqrt{2}\right){\left(1-\alpha \right)}^{2}$$

The first and second order coefficients of $\tilde{R}$ are positive, and the constant coefficient is non-negative for all α∈[0,1]. Thus, $b_{2}>0$ for $\tilde{R}>0$ and for all α∈[0,1], or equivalently, for all $R>(\sqrt{2}-1)/2$. In this case, $a_{2}>0$ also, which then implies that $p({\tilde{\epsilon }}_{0}+{\tilde{\epsilon }}_{1})>0$, as required.

Thus, we have proved that $p({\tilde{\epsilon }}_{0}+{\tilde{\epsilon }}_{1})>0$ using Condition (61). However, even if $b_{2}<0$, there are still many other positive terms in $p({\tilde{\epsilon }}_{0}+{\tilde{\epsilon }}_{1})$, and so, it may be the case that Condition (61) is not necessary. We note that the minimum of $b_{2}$ in the region R≥0, α∈[0,1] occurs at R=0, α=1. Substituting R=0, α=1 into the cubic polynomial in *P* in (71) gives:

$$\left(a_{3}P^{3}+a_{2}P^{2}+a_{1}P+a_{0}\right){|}_{R=0,\alpha =1}=B^{2}\left(1+P\right)\left(B+C-CP\right)$$

Using assumption (42), this is positive. Moreover, expanding the cubic polynomial in a Taylor series about the point (R,α)=(0,1), we find that the first order terms are positive, and so, for sufficiently small R>0 and α<1, the cubic polynomial in *P* will be positive. However, this does not guarantee that it is positive for all R>0 and α∈[0,1], although we conjecture that this is in fact the case.

Now, the two branches of solutions (70) occur on either side of the gap in ϵ, and so, one branch exists for $\epsilon <{\tilde{\epsilon }}_{0}+{\tilde{\epsilon }}_{1}$ and the other for $\epsilon >{\tilde{\epsilon }}_{0}+{\tilde{\epsilon }}_{1}$. The left-hand branch is therefore the only branch of infected solutions for $\epsilon <{\tilde{\epsilon }}_{0}+{\tilde{\epsilon }}_{1}$, and so, the two bifurcating branches of infected solutions arising from the two bifurcations described above must be part of this single branch.

Thus, there is a single branch of infected solutions that connects the two bifurcation points, as claimed. There must also be a limit point on this branch, which occurs for $\epsilon >\epsilon_{2}$.

**Figure 5:**
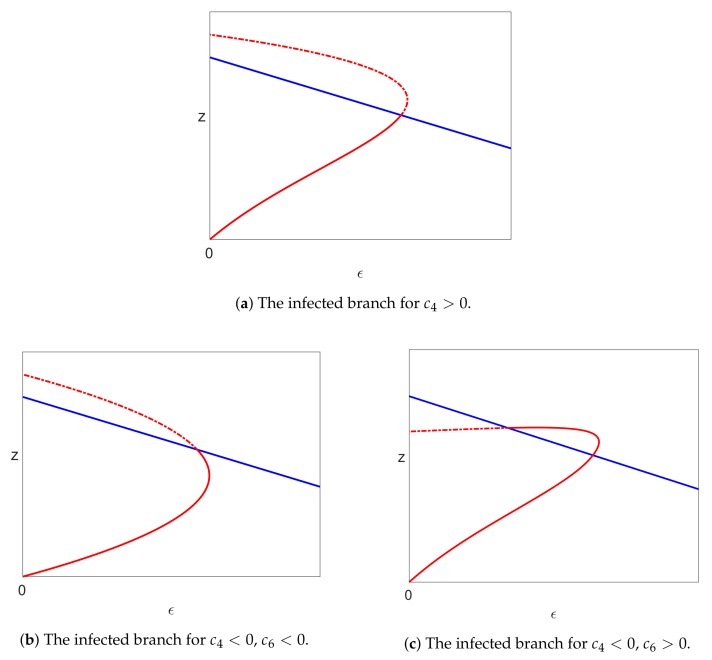
The pure infection (blue) and infected (red) steady state branches. Note that the dashed-dotted lines indicate invalid solutions.

The only steady state solutions with x=0 are the uninfected or pure infection solutions. Thus, all other solutions, in particular the infected solutions, have x≠0. In the case of $c_{4}>0$ (Figure 4 (top)), the only way that the valid infected branches arising from the two bifurcations can connect on a curve with a single limit point is for the limit point to occur on the invalid solutions after the bifurcation on the pure infection branch, as shown in Figure 5a. When $c_{4}<0$ (Figure 4 (middle, bottom)), the valid infected solution emanates from the pure infection branch to the right of the bifurcation point, and the only way that this can connect to the bifurcation on the uninfected branch at ϵ=0 is for there to be a limit point in between the two bifurcations.

We note that the left branch of (69) exists for all δz, but does not exist for all *z*. As δϵ→−∞ (β→±∞), the left branch of the hyperbola asymptotes to the straight lines $\delta z=\pm \sqrt{h_{1}/h_{2}}\;\delta \epsilon$. Substituting these into z(β) given by (70), we see that *z* converges to the constant values:

$$z=z_{0}\pm \sqrt{h_{1}/h_{2}}=\frac{1}{2CR}\left(1\pm \sqrt{1+4CPR(1+R)/(\alpha B)}\right)$$

as ϵ→−∞. Clearly one of these asymptotes is negative and the other positive, as we would expect. ☐

Finally, we recall the assumption we made in Theorem 4 that *D* is defined by (40). If this is not the case, then the same method described above can be used to derive Equation (69), and $z_{0}$, ${\tilde{\epsilon }}_{0}$, $h_{1}$ and $h_{2}$ are unchanged. However, the other coefficients now involve the parameter *D*. In this case, it is not possible to determine the sign of the coefficient $h_{0}$ in (69). As long as $h_{0}$ remains positive, the same picture as described above will hold qualitatively. However, if $h_{0}$ changes sign and becomes negative, then the structure of the solutions of (69) changes, so that there are no limit points occurring. Therefore, if (40) does not hold, then there is an extra condition $h_{0}>0$ that is required to give the same solution structure.

### 5.4. Steady States (S>0)

We have studied in detail the solutions of our model in the special case when S=0, which is when there is no production of hepatocytes from stem cells. When *S* is small and positive, this will result in a small perturbation of these solutions, and we now consider this case.

#### 5.4.1. Bifurcation on the Uninfected Branch

When S>0, the uninfected steady state (43) and the bifurcation point that is found by solving (48) between the uninfected and infected steady state branches both remain the same. Therefore, the condition (40) again ensures that this bifurcation point occurs at ϵ=0.

#### 5.4.2. Bifurcation on the Pure Infection Branch

When S>0, the state of pure infection no longer exists since there is a second mechanism for generating healthy hepatocytes due to stem cell production. Thus, for small values of *S*, the bifurcation involving the infected and pure infection branches will unfold. There are two possible ways that a transcritical bifurcation can unfold, depending on the sign of the perturbation term.

When S>0, the bifurcation equation (60) in *x* simply gains an extra term Sy where *y* is given by (58) and so is given by:

(72) $$h\left(x,\delta \epsilon \right)=S\left(y_{p}+O\left(x,x^{2},x\delta \epsilon \right)\right)+c_{3}x\delta \epsilon +c_{4}x^{2}+xO\left(x,\delta \epsilon \right)$$

where $\delta \epsilon =\epsilon -\epsilon_{2}$. The constant term is the only term needed to determine the unfolding of a transcritical bifurcation [21]. Limit points for this equation are found by solving $h\left(x,\delta \epsilon \right)=h_{x}\left(x,\delta \epsilon \right)=0$ and are then given by:

$$x^{2}=\frac{Sy_{p}}{c_{4}}+O\left(S^{2}\right)$$

Since S>0, we see that for sufficiently small *S*:

- if $c_{4}<0$, then no limit points exist;
- if $c_{4}>0$, then there are two limit points at $x=\pm \sqrt{Sy_{p}/c_{4}}$.

**Figure 6:**
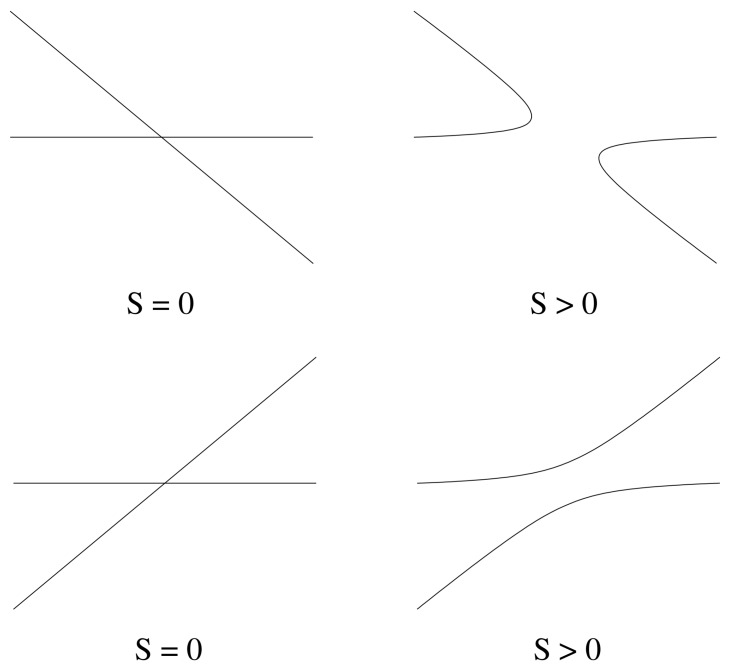
Unfolding of the transcritical bifurcation given by (72) in the (ϵ,x) plane for $c_{4}>0$ (top) and $c_{4}<0$ (bottom).

We recall that $c_{3}>0$, and so, the sign of $c_{4}$ also determines the slope of the bifurcating branch (see Figure 4). The unfolding of the bifurcation in these two cases is shown in Figure 6. We note that when S>0, in both cases, one of the branches locally has x<0, and one has x>0; so, there is only one valid branch of solutions.

**Figure 7:**
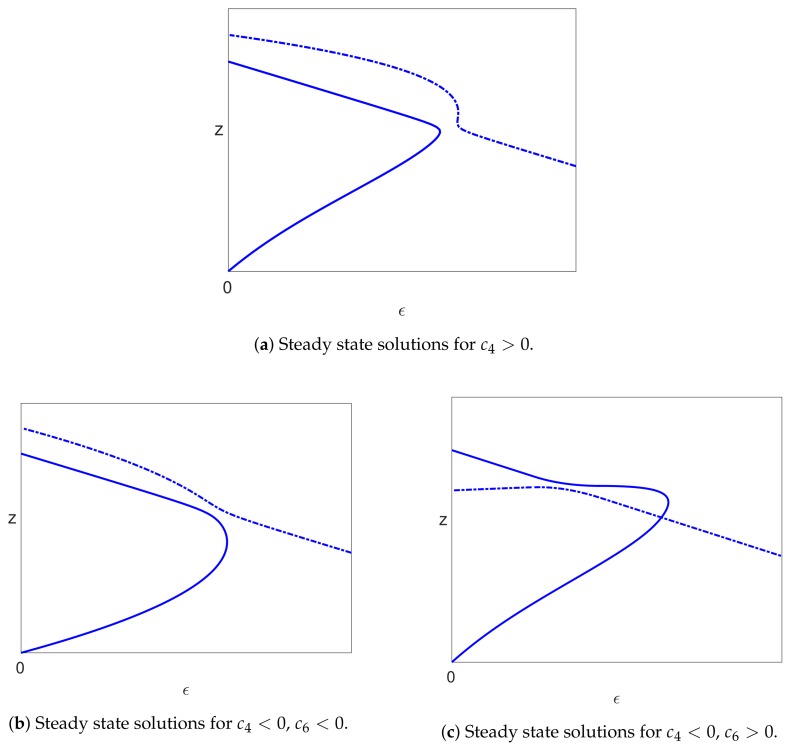
Steady state solutions where the bifurcation between the infected and pure infection branches has been unfolded as S>0, assuming that $\epsilon_{2}>0$. The dashed-dotted lines are invalid solutions as x<0.

When $\epsilon_{2}>0$, the unfolding of the solutions in Figure 5 is shown in Figure 7.

#### 5.4.3. Infected Branch of Solutions

For the infected solution branch away from the bifurcation points, the implicit function theorem implies that a small perturbation in the equations due to S>0 results in an O(S) perturbation in the infected solutions, and so, there is only a small perturbation in this branch of solutions. Thus, we see that in all cases, the form of the infected steady states is qualitatively similar since the branch of infected states emanates from the trivial solution at the bifurcation point at ϵ=0 and increases with ϵ, goes round a limit point and then decreases, as shown in Figure 7. Moreover, this is the only valid branch of infected steady state solutions.

#### 5.4.4. The Case of $\epsilon_{2}<0$

Finally, we consider one more situation, namely when condition (56) does not hold. Thus, we now assume that:

(73) BP−CD(1+R)(1+D(1+R))<0

**Figure 8:**
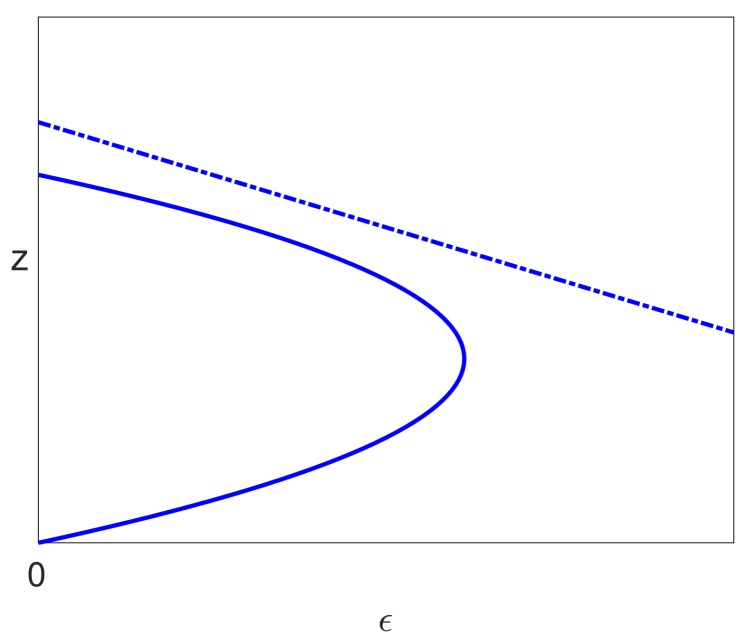
The steady state solutions when (73) holds and S>0. The dashed-dotted line consists of invalid solutions as x<0.

This implies that $\epsilon_{2}<0$ by Lemma 3. and so, the bifurcation point on the pure infection branch with S=0 occurs for negative ϵ. The infected branch of solutions still has a limit point in this case, but there is no connection between these two branches in our range of interest given by ϵ∈[0,1). Moreover, the pure infection steady state with no treatment (ϵ=0) is unstable, since Condition (56) was required to ensure stability. In this case, the unfolding of the bifurcation when S>0 occurs in the same way, but outside our range of interest, and the pure infection branch becomes invalid. The solutions in this case are shown in Figure 8. We note that the valid solution branch is similar in this case as in the previous cases considered, with a single limit point occurring.

## 6. Stability

**Figure 9:**
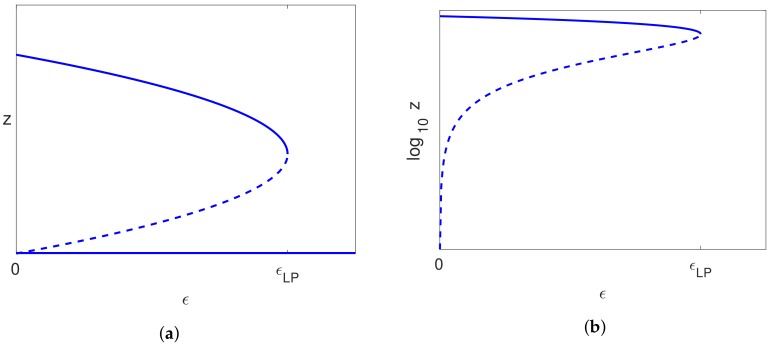
The bifurcation diagram, where solid lines indicate stable solutions and dashed lines indicate unstable solutions. Note that the vertical scale is either (**a**) *z* or (**b**) $\log_{10}z$.

All the cases considered above, with Assumptions (39) and (40), give rise, when S>0, to an uninfected steady state branch x=1, y=z=0 together with a branch of infected steady state solutions that bifurcates from ϵ=0 and has a single limit point. We have also seen that the uninfected branch of solutions is stable for ϵ∈(0,1), and this implies that the bifurcating branch of infected solutions initially has one unstable eigenvalue together with two stable eigenvalues. The only way that the infected branch can become stable again is if the unstable eigenvalue passes back through zero, which would correspond to a limit point. We have also ascertained that there is precisely one limit point on the branch of infected solutions (for sufficiently small *S*). Since the solutions past the limit point are stable when S=0, they will still be stable for small S>0, and so, the one unstable eigenvalue must pass through zero at the limit point to give a stable branch of solutions. Thus, the bifurcation diagram for S>0 (and sufficiently small) is as shown in Figure 9a.

The other possibility is that the two stable eigenvalues along the unstable section of the infected branch could collide and become complex and then cross the imaginary axis in a Hopf bifurcation. They would then have to cross back again in a reverse Hopf bifurcation before the limit point in order for the solution to stabilise after going round the limit point. However, to determine whether or not such Hopf bifurcations occur analytically from the model is a very challenging problem. It is also of little interest, since any bifurcating periodic orbits would be unstable.

## 7. Comparison with the Neumann/Dahari Models

We now compare the predictions from our new model of HCV infection with those of the Neumann and Dahari models that we reviewed in Section 2. Our model is similar to previous models in that it involves the three variables *T*, *I* and *V*. However, the steady state solutions, and therefore the dynamics, of the new model are quite different from previous models in several ways. These differences result in different predictions for the dynamics of the infection during treatment and suggest possible different treatment regimes.

**Figure 10:**
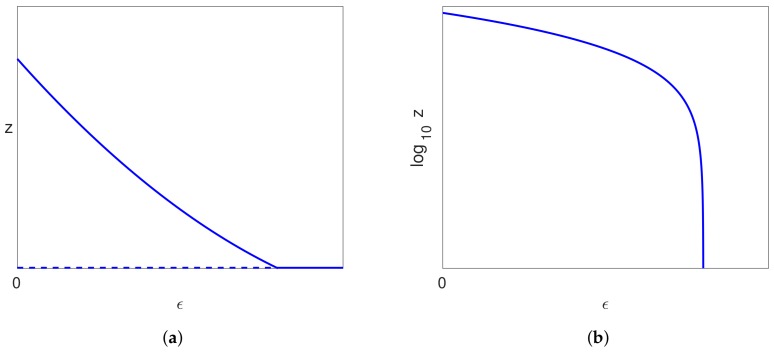
A typical bifurcation diagram for the Neumann/Dahari models, where solid lines indicate stable solutions and dashed lines indicate unstable solutions. Note that the vertical scale is either (**a**) *z* or (**b**) $\log_{10}z$.

A typical bifurcation diagram for the Neumann/Dahari models is shown in Figure 10a, which we compare with the bifurcation diagram for our model, as shown in Figure 9a. We also note that experimental data are plotted with $\log_{10}V$ on the vertical axis rather than *V*, and so, we also show the bifurcation diagrams plotted for $\log_{10}z$ in Figure 9b and Figure 10b. We also note from (26) that $\log_{10}V=\log_{10}z+k$ where $k=\log_{10}\left(r_{T}T_{\max}/\left(r_{T}+d\right)\right)$, and so, the non-dimensionalisation only results in a shift on the vertical axis.

We now make a number of comparisons between these models.

- For the Neumann/Dahari models, treatment will only be effective once the treatment factor ϵ exceeds a critical value determined by the bifurcation point, regardless of the viral load when treatment commences. For our model, if the viral load is close to the infected steady state before treatment starts, then similarly, the treatment factor ϵ must exceed the critical value $\epsilon_{LP}$ determined by the limit point for the treatment to be effective. However, if the infection is caught and treated in the early stages, while the viral load is still relatively low, then our model predicts that a lower drug dose, with a corresponding smaller value of the treatment parameter ϵ, will be effective.
- As mentioned previously, once treatment is stopped, the prediction of the Neumann/Dahari models is that the infection will take hold again unless the infected hepatocytes and virus have been completed eliminated during treatment. The prediction from our model, if the bifurcation point on the uninfected solution branch occurs at a negative value of ϵ ($\epsilon_{0}<0$), is that the body will be able to eliminate a small amount of infected hepatocytes and virus cells without further treatment once their levels have been reduced sufficiently. On the other hand, if the bifurcation point on the uninfected branch occurs at a positive value of ϵ ($\epsilon_{0}>0$), then our model predicts in this case that the infection will take hold again on cessation of treatment unless the infected hepatocytes and virus cells have been completely eliminated during treatment. However, our model also predicts that continuing with a low level of drug treatment, corresponding to a small value of ϵ, will stop the infection recurring in this case.
- The Neumann/Dahari models suggest that treatment will only be effective if the treatment parameter ϵ is greater than the critical value during the whole period of treatment, which is the way that patients are generally treated in practice. Our model suggests that the drug dose could be reduced as treatment progresses and that this will still be effective, provided that it is not reduced too far too quickly. If this is indeed the case, it could save some of the costs of treatment, and a lower drug dosage may also mean a reduction in side effects, which would benefit the patient.

## 8. Description of Observed Viral Load Profiles

The usual approach with a new model is to fit it to data in order to show that there are values of the model parameters that give a good fit to these data. This is a useful approach, but does not give any insight into the mechanisms involved in the different cases. Thus, we now consider many of the observed behaviours of the viral load under treatment that are reported in the literature and show how our model can explain these observations. This also helps to explain the possible mechanism associated with the observations in some cases. In the next section, we then fit our model to four datasets to show that this can also be done in practice.

A number of different viral load profiles were reported in [5], and we first consider these. However, we also consider various other observations in the literature. In this section, we define $\epsilon^{*}\in \left(0,1\right)$ to be the value of the treatment parameter in the model, which may of course vary for different patients.

### 8.1. Sustained Virologic Response

Sustained virologic response is where the viral load rapidly decreases and is undetectable at the completion of treatment at 24 weeks [5]. For our model, this is easily realised by having $\epsilon_{LP}<\epsilon^{*}$ so that the trajectory in our model converges to the only available uninfected steady state, which is stable provided that $\epsilon_{0}<\epsilon^{*}$. To see this rapid one phase decline, it is likely that treatment commenced not too long after infection, since a delay in treatment is often associated with biphasic or triphasic decline of the viral load (see Section 8.6). Once treatment is stopped, the viral load remains undetectable, and this will be the case provided that $\epsilon_{0}<0$, or equivalently BP<D(B+C) (see Lemma 1 and the discussion in Section 3.5).

### 8.2. Relapse

Relapse is similar to sustained virological response (SVR) during treatment, in that there is a rapid decline in viral load. However, once treatment stops, the patient relapses as the viral load increases back to pre-treatment levels. This relapse occurs if $\epsilon_{0}>0$ as discussed in Section 3.5. In this case, continuing with a lower drug dose may be sufficient to keep the viral load under control.

### 8.3. Partial Virologic Response

There is a partial virologic response (PVR) if an initial decrease in viral load is followed by an increase during treatment. This could be explained by our model if $\epsilon^{*}<\epsilon_{LP}$ and with the viral load quite high before the start of treatment. In this case, the trajectory of the model would converge to the infected steady state at $\epsilon^{*}$, and it is quite possible that it could initially overshoot this steady state and then return back to it.

### 8.4. Breakthrough

Breakthrough is similar to PVR, except that at some point during treatment, the viral load is undetectable before increasing again during treatment. Thus, the mechanism would be similar to that described for PVR, but with the infected steady state occurring at a lower viral load, so that there is a larger initial drop in the viral load, below the level of detection, before increasing again back up to the infected steady state.

### 8.5. Null Response

Some patients show no significant reduction in viral load under treatment. This could occur when $\epsilon^{*}<\epsilon_{LP}$ and where the infected steady state at $\epsilon^{*}$ is similar to that at ϵ=0. This could occur when $c_{4}<0$ and $c_{6}>0$, as shown in Figure 7c.

### 8.6. Biphasic and Triphasic Decline

A common observation of the viral load during treatment is that there is biphasic decline [3], although triphasic decline, with a flat region between two declining phases, has also been observed [3,4]. We now show that both of these patterns of decline in the viral load under treatment can occur in our model.

We assume that *S*, the parameter associated with the generation of hepatocytes due to stem cells, is small (and positive). When there is no treatment (ϵ=0), the infected steady state is then given by:

$$\begin{array}{ccc} x & = & \frac{C}{BP-CD(1+R)(1+D(1+R))}S+O\left(S^{2}\right)\\ y & = & y_{p}+O\left(S\right)\\ z & = & z_{p}\left(0\right)+O\left(S\right) \end{array}$$

where $y_{p}$ and $z_{p}\left(0\right)$ are the pure infection steady states given by (44). We assume that (56) holds so that this solution is both valid (as x>0) and stable.

If we assume that a patient has been infected for a long time before treatment, then this implies that at the start of treatment, x(0), y(0) and z(0) will be close to these steady state values. We therefore now assume that x=O(S), and so, we set $x=S\tilde{x}$. Substituting for *x* in (29)–(31) and taking only the leading order terms in *S* gives the reduced equations:

(74) $$\begin{array}{ccc} {\tilde{x}}^{\prime } & = & y+\frac{\tilde{x}}{y}-\tilde{x}-\left(1-\alpha \epsilon \right)B\tilde{x}z \end{array}$$

(75) $$\begin{array}{ccc} y^{\prime } & = & \frac{1}{1+R}\left(1-y\right)-Dy \end{array}$$

(76) $$\begin{array}{ccc} z^{\prime } & = & (1-\epsilon )Py-Cz \end{array}$$

We note that equation (75) is a linear equation in *y* with stable steady state $y=y_{p}$. Since we are assuming that y(0) is close to this steady state, dynamically, *y* will continue to converge towards the steady state and is not influenced by either *x* or *z*.

Equation (76) has the stable steady state $z=z_{p}\left(\epsilon \right)$. Once treatment starts with $\epsilon =\epsilon^{*}$, this steady state drops from $z_{p}\left(0\right)$ to $z_{p}\left(\epsilon^{*}\right)=\left(1-\epsilon^{*}\right)z_{p}\left(0\right)$. Since *y* is approximately constant, this implies at the start of treatment that *z* will decay exponentially towards the new steady state value. If we assume that $y=y_{p}$, then the solution of (76) is:

(77) $$z=z_{p}\left(\epsilon^{*}\right)+\left(z\left(0\right)-z_{p}\left(\epsilon^{*}\right)\right)e^{-Ct}$$

This is the observed first phase of rapid decline in the viral load. We note that Equation (74) can be written as:

$${\tilde{x}}^{\prime }=y+\frac{\tilde{x}}{y}-\tilde{x}-B\tilde{x}z+\left(\alpha B\tilde{x}z\right)\epsilon$$

Thus, the effect of increasing ϵ at the start of treatment is to increase ${\tilde{x}}^{\prime }$, and this will result in $\tilde{x}$ increasing also.

Once *z* has dropped to close to the steady state $z_{p}\left(\epsilon^{*}\right)$, and assuming that *y* is also close to $y_{p}$, then $y^{\prime }=O\left(S\right)$ and $z^{\prime }=O\left(S\right)$, and so, *y* and *z* evolve on a slow time scale. Thus, they will remain approximately constant for a time of O(1/S). This is the flat middle phase in the triphasic decline.

Eventually, the O(S) terms in the *y* and *z* equations will result in *y* and *z* being displaced from their leading order steady states, and the third phase decline towards the uninfected steady state will start. This rate of decline is determined by the eigenvalue of the Jacobian matrix evaluated at the uninfected steady state given in (47) that is closest to zero. Clearly, one eigenvalue is $\lambda_{1}=-1$, and this is unlikely to be the closest to zero. The other two eigenvalues are found from the lower 2×2 matrix. The characteristic equation, which has to be solved for these eigenvalues, is given by:

(78) $$p\left(\lambda \right)=\lambda^{2}+\left(\left(1-\alpha \epsilon \right)B+C+D\right)\lambda -BP\left(1-\epsilon \right)\left(1-\alpha \epsilon \right)+D\left(\left(1-\alpha \epsilon \right)B+C\right)=0$$

Assuming that $\epsilon^{*}>\epsilon_{0}$, then the uninfected steady state is stable, and so, the two solutions of this characteristic equation are both negative. Suppose that these solutions are $-\lambda_{1}$ and $-\lambda_{0}$ with $-\lambda_{1}<-\lambda_{0}<0$. In this case, the rate of decline to the uninfected steady state is determined by the eigenvalue closest to zero, which is $-\lambda_{0}$. The third phase decline is generally observed to be slower than the first phase, and this will be the case if $C>\lambda_{0}$. Now, p(λ)<0 if and only if $-\lambda_{1}<\lambda <-\lambda_{0}$. Thus, if p(−C)<0, then this implies that $-C<-\lambda_{0}$ as required. Now:

$$p\left(-C\right)=-\left(1-\alpha \epsilon^{*}\right)B\left(C+\left(1-\epsilon^{*}\right)P-D\right)$$

Clearly, this is negative if C+(1−ϵ)P>D, and so, this is the condition that ensures that the third phase decline is slower than the first phase.

Thus, we have shown that our new model can exhibit triphasic decline of the viral load, as is seen in the data. This analysis also suggests that this pattern of decline is associated with late commencement of treatment so that the initial conditions for the model are close to the infected steady state. This is also the conclusion reached by Dahari et al. [3].

Biphasic decline is similar to triphasic decline, but where the middle flat phase is very short. The first order term in *S* in Equations (75) and (76) is $\pm S\left(1-\alpha \epsilon^{*}\right)B\tilde{x}z$, and so, anything that increases the magnitude of this term will reduce the length of the middle phase. This includes a larger value of *S* or *B* or a larger value of $z_{p}\left(\epsilon^{*}\right)$. We note that an increase in *B* will also result in a more rapid increase in *x*, which will also help to shorten this phase. Thus, our model can also exhibit biphasic decline of the viral load.

### 8.7. Initial Increase in Viral Load

Hsu et al. [22] reported that in some patients, there is an initial increase in viral load when treatment is started and that this initial increase is associated with a higher likelihood of achieving SVR. They used different models to investigate this behaviour and concluded that a modification of the Neumann model gave the best fit provided that c=δ and η=1, which implies that the treatment always provides a complete block on de novo infection. Guedj et al. [23] questioned these unrealistic assumptions and the analysis in [22] and suggested that the initial increase in viral load was due to the infected steady state not having been reached so that viral loads were increasing before the start of therapy. They also suggested that the correlation between the initial increases and SVR was an indication of the effectiveness of the therapy. Rong and Perelson [24] also considered this effect in their multiscale model for direct acting antiviral agents. Their model always showed a small initial increase in viral load, even when starting from steady state. Of course, this result cannot be directly compared with the experimental results of Hsu et al. [22] since these results were obtained for patients treated with interferon (IFN) and ribavirin (RBV) only.

To understand this effect from our model, we express (31) as:

(79) $$z^{\prime }=Py-Cz-Bxz-\epsilon \left(Py-\alpha Bxz\right)$$

Without treatment (ϵ=0), we expect *z* to be increasing towards the infected steady state, and so $z^{\prime }>0$, which implies that:

(80) Py−Cz−Bxz>0

We note that:

Py−αBxz=(Py−Cz−Bxz)+(Cz+(1−α)Bxz)>0

assuming that (80) holds and using (37).

If we assume that the infected steady state $\left(x_{i},y_{i},z_{i}\right)$ has been reached before the start of treatment, then at t=0, we have:

$$z^{\prime }\left(0\right)=-\epsilon^{*}\left(Py_{i}-\alpha Bx_{i}z_{i}\right)=-\epsilon \left(Cz_{i}+\left(1-\alpha \right)Bxz\right)$$

which is negative, and so, we have an immediate decline in viral load. However, if the steady state has not been reached before treatment commences, then with (x,y,z)=(x(0),y(0),z(0)) at the start of treatment, we have:

$$z^{\prime }\left(0\right)=Py\left(0\right)-Cz\left(0\right)-Bx\left(0\right)z\left(0\right)-\epsilon^{*}\left(Py\left(0\right)-\alpha Bx\left(0\right)z\left(0\right)\right)$$

which is the difference of two positive terms, and so could be negative or positive. Larger values of $\epsilon^{*}$ are more likely to give $z^{\prime }\left(0\right)<0$, and so, the effectiveness of treatment does not explain the correlation between the initial increase and SVR, as suggested in [23]. We propose an alternative explanation for this correlation.

Soon after infection, the viral load will be increasing rapidly while *y* and *z* are both quite small, and so, the effect of the treatment will be small also, so that $z^{\prime }\left(0\right)>0$, thus giving an initial increase in viral load. However, if the start of treatment is delayed and the system is getting towards the infected steady state, then $z^{\prime }\left(0\right)$ will be quite small before treatment commences. However, near the steady state, Py(0) will be relatively large, so that the net effect gives $z^{\prime }\left(0\right)<0$ once treatment starts. Thus, according to this model, an initial increase in viral load is associated with early initiation of treatment, which is likely to correlate with a higher rate of SVR, as reported in [22].

### 8.8. Direct Acting Antiviral Agents

Recently, new direct acting antiviral (DAA) agents have become available, which are more effective than treatment with IFN and RBV alone. If DAAs are used as a monotherapy, then it is found that drug-resistant virus cells quickly form, rendering the treatment ineffective; so, they are used in combination with IFN and RBV, and this combination is found to be highly effective [25]. More complex models for the action of DAAs have been proposed [26]. It has been found that treatment that includes DAAs has notable differences compared to treatment with only IFN and RBV, which include (i) a more rapid and longer first phase decline and (ii) a more rapid second phase decline [25].

We note that the action of a DAA is essentially to further block viral replication, although by different mechanisms than the older drugs [27]. The simplest way that this can be modelled is to increase the parameter ϵ in (31). If it is also assumed that the DAAs do not have any additional effect in reducing the rate of production of infected cells (parameter η in Equations (19)–(21)), then an increase in ϵ must be accompanied by a corresponding reduction in the parameter α given in (27), in order to keep η=1−αϵ>0 constant. We claim that this simple change is sufficient to explain both of the above observed effects, which we now justify.

**Figure 11:**
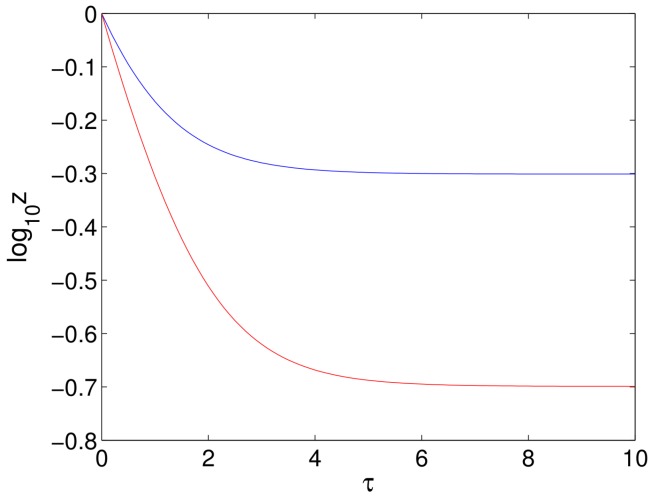
The decline in viral load given by (77) with $z_{p}\left(0\right)=1$, C=1 and $z_{p}\left(\epsilon^{*}\right)=0.5$ (blue) or $z_{p}\left(\epsilon^{*}\right)=0.2$ (red).

(i) It has been noted that the first phase decline when treating with DAAs is both longer and faster than when using IFN and RBV. Since the rate of the first phase decline is essentially given by the parameter *C* (or *c* for the original equations), it has been suggested that both ϵ and *c* should be increased in the models for treatment with DAAs [25]. However, we claim that an increase in ϵ alone is sufficient to produce a longer and faster first phase. To see this, we consider the decline in viral load during the first phase that is given in (77). For this solution, we find that the initial rate of decline is:
  $$\begin{array}{ccc} {\left.\frac{d(\log_{10}z)}{d\tau }\right|}_{\tau =0} & = & -\left(1-\frac{z_{p}\left(\epsilon^{*}\right)}{z_{p}\left(0\right)}\right)C\log_{10}e\\ & = & -\epsilon^{*}C\log_{10}e \end{array}$$
  since $z_{p}\left(\epsilon^{*}\right)=\left(1-\epsilon^{*}\right)z_{p}\left(0\right)$, and so, the initial slope increases as the treatment factor $\epsilon^{*}$ increases, with maximum slope only being achieved when $\epsilon^{*}=1$ (which corresponds to $z_{p}\left(\epsilon^{*}\right)=0)$. Clearly, the steady state $z_{p}\left(\epsilon^{*}\right)$ is also reduced as $\epsilon^{*}$ is increased. These two effects result in an increase in the length of the decline in the viral load together with a more rapid decline. This is illustrated in Figure 11.
(ii) The second observation made for DAAs is that the second phase is also faster than that for treatment with IFN and RBV. We have seen in Section 8.6 that for our model, the rate of decay in the second phase is proportional to $e^{-\lambda_{0}t}$ where $-\lambda_{0}<0$ is the solution of the characteristic equation (78) that is closest to zero. With our assumption that η=1−αϵ is constant, (78) becomes:
  (81) $$p\left(\lambda \left(\epsilon \right)\right)=\lambda {\left(\epsilon \right)}^{2}+\left(\eta B+C+D\right)\lambda \left(\epsilon \right)-\eta BP\left(1-\epsilon \right)+D\left(\eta B+C\right)=0$$
  Differentiating this equation with respect to ϵ and solving for $\lambda^{\prime }\left(\epsilon \right)$ gives:
  $$\lambda^{\prime }\left(\epsilon \right)=-\frac{\eta BP}{\left(\eta B+C+D+2\lambda (\epsilon )\right)}$$
  and evaluating at $\epsilon =\epsilon^{*}$ gives:
  (82) $$\lambda^{\prime }\left(\epsilon^{*}\right)=-\frac{\eta BP}{\left(\eta B+C+D-2\lambda_{0}\right)}$$
  for $\lambda \left(\epsilon^{*}\right)=-\lambda_{0}$. The sign of the denominator is not clear. However, substituting λ=−(ηB+C+D)/2 into p(λ) given by (81) gives:
  $$p\left(-\frac{1}{2}\left(\eta B+C+D\right)\right)=-\left(\left(1-\epsilon \right)\eta BP+\frac{1}{4}{\left(\eta B+C-D\right)}^{2}\right)<0$$
  using (38) and the assumption that η>0. The quadratic coefficient of λ(ϵ) in (81) is positive, and so, p(λ(ϵ)) is only negative between the two roots $-\lambda_{1}$ and $-\lambda_{0}$, which implies that:
  $$-\lambda_{1}<-\frac{1}{2}\left(\eta B+C+D\right)<-\lambda_{0}$$
  It follows from this that the denominator of (82) is positive, and so, $\lambda^{\prime }\left(\epsilon^{*}\right)<0$. Hence, if ϵ is increased from $\epsilon^{*}$, then the eigenvalue $-\lambda_{0}$ will decrease to first order, thereby increasing the rate of the second phase decline.

Thus, the observed effects of direct acting antiviral agents, in association with IFN and RBV, can be included in our model by simply keeping 1−αϵ constant and reducing ϵ.

## 9. Data Fitting

We claimed in Section 8 that our new model is capable of generating many of the observed profiles of viral load under treatment. We now fit our model to some data in order to demonstrate that this is indeed the case.

For this data fitting, we use the model equations in dimensional form given by (19)–(21). We first note that it is not possible to fit all of the parameters in the model. In particular, the parameters η and β always occur in the combination (1−η)β, and so, during treatment, we can only hope to determine this single quantity from the data. Similarly, ϵ and *p* occur in the combination (1−ϵ)p, and so again, we can only determine this single quantity during treatment.

We also observe that the five parameters $r_{T}$, $r_{I}$, $T_{\max}$, *d* and δ occur only in the four groups $r_{T}T_{\max}$, $r_{I}T_{\max}$, $r_{T}+d$, $r_{I}+\delta$, and so, it will not be possible to identify all five of these parameters from the data. Instead of using these four parameter groups, we use $r_{T}T_{\max}$, $r_{T}+d$, *R* and *D*, where *R* and *D* are non-dimensional parameters defined in (32). We write Equations (19)–(21) in terms of these parameters as:

(83) $$\begin{array}{ccc} \dot{T} & = & sI+\frac{r_{T}T_{\max}T}{T+I}-\left(r_{T}+d\right)T-\beta^{*}VT \end{array}$$

(84) $$\begin{array}{ccc} \dot{I} & = & \frac{1}{1+R}\left(\frac{r_{T}T_{\max}I}{T+I}-\left(r_{T}+d\right)I\right)-D\left(r_{T}+d\right)I+\beta^{*}VT \end{array}$$

(85) $$\begin{array}{ccc} \dot{V} & = & p^{*}I-cV-\beta^{*}VT \end{array}$$

where:

$$\beta^{*}=\left(1-\eta \right)\beta ,\;\;\;\;p^{*}=\left(1-\epsilon \right)p$$

Values of the parameter groups $r_{T}T_{\max}$ and $r_{T}+d$ will be found by fitting the model to data. We note that by including the non-dimensional parameter *R*, and requiring it to be positive, ensures that the conditions $0<r_{I}/r_{T}<1$ are satisfied. Moreover, requiring D>0 is consistent with the conditions $r_{I}<r_{T}$ and d<δ, but does not guarantee that they hold. We also note that it is not possible to determine whether Condition (39) holds since the parameter β cannot be determined. The sign of $\epsilon_{0}$, the value of ϵ at which the bifurcation on the uninfected branch occurs (see Lemma 1), cannot be determined either since β and *p* cannot be determined.

The initial value V(0) will be taken from the data and the other initial values, T(0) and I(0), will be regarded as unknown parameters. Thus, there is a total of 10 parameters and initial values to be found by fitting the model Equations (83)–(85) to the data, all of which are required to be positive. These parameters are found using the method of least squares in which the sum of the squares of the differences between the log of the viral load data points and the log of the viral load predicted by the model at the given time points is minimised. This task was performed using Matlab with the differential equations described in terms of the log of each of the variables.

**Figure 12:**
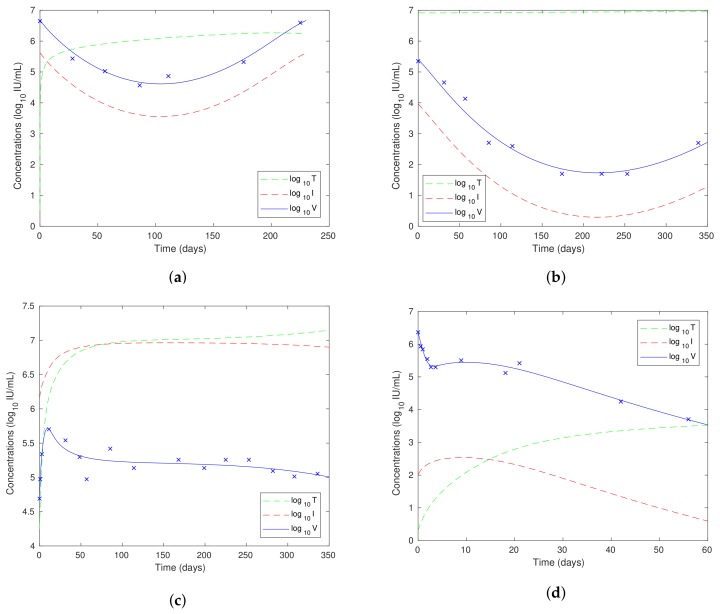
Plots of the viral load *V* (blue), healthy hepatocytes *T* (green) and infected hepatocytes *I* (red) against time fitted to the viral load datasets for (**a**) partial virologic response (PVR), (**b**) breakthrough, (**c**) null response, (**d**) triphasic.

We considered various datasets that show partial virologic response (PVR), breakthrough, null response and triphasic behaviour. The first three datasets are taken from [5], while the last one is taken from [6]. We fitted our model to the data during treatment only. The data together with the fitted curve V(t) and the predicted curves T(t) and I(t) from the model for each case are shown in Figure 12. The parameter values used are given in Table 1. These should not be regarded as definitive parameter values since very similar fits to the viral load data can be found using quite different sets of parameter values.

We did not consider data that shows sustained virologic response (SVR) since these datasets typically contain only two data points before the viral load goes below the lower limit of quantification (see [5]), and there will be many parameter combinations that will fit the two data points. The model can of course show this behaviour for appropriate parameter values.

For the breakthrough data, we note that the three lowest data points are recorded as 50 IU/mL, which is the lower limit of quantification, and so, the actual values of the viral load will be lower than this. However, we do not know the correct values of the viral load, and so, for the data fitting, we kept the outer two of these values, but ignored the middle value.

We now make a number of observations regarding these results.

- In the PVR, breakthrough and triphasic cases, we have I(t)<V(t) for all *t*, but in the null response case, I(t) is significantly higher than V(t).
- The initial viral load is highest in the PVR case, and we predicted in Section 8.3 that PVR would be associated with a high initial viral load.
- In the null response case, it is interesting to observe that the fitted viral load *V* and infected hepatocyte concentration *I* both reduce towards zero, but very slowly. The start of the decline in these variables can be observed from around 300 days in Figure 12c. At 1000 days after the start of treatment, the predicted values are $\log_{10}I\left(1000\right)=5.1202$ and $\log_{10}V\left(1000\right)=2.8515$.
- In Section 8.6, we stated that triphasic decline might be expected when the patient has been infected for a long time before treatment, which means that the viral load will be high while the healthy hepatocyte concentration will be very low. This is precisely the situation observed in Figure 12d.
- In Section 8.7, we showed that an initial increase in viral load at the start of treatment is possible, and we see this in the null response case.
- We saw in Section 3.1 that the regeneration rate for a healthy liver is $1.15\times 10^{-2}$ day${}^{-1}$ for females and $1.11\times 10^{-2}$ day${}^{-1}$ for males. This is effectively the parameter $r_{T}+d$ in our new model. The value of this parameter when fitted to data is a little lower than this for the PVR and breakthrough cases and is slightly higher in the null response and triphasic cases.
- The condition (33) for the solution to be bounded for all t≥0 in terms of the parameters we are using here is given by:
  $$\min\left(s,p^{*}\right)\left(1+R\right)-\left(r_{T}+d\right)\left(1+D\left(1+R\right)\right)<0$$
  The term on the LHS for each of the fits to the data is given by: PVR: −0.1790; breakthrough: −2003; null response: −0.1753; triphasic: −0.2500. All of these values are negative, which ensures that each of the solutions exists and is bounded for all time by Theorem 3.

## 10. Conclusions

We have proposed a new mathematical model of HCV infection, which involves the same three variables (concentrations of healthy and infected hepatocytes and of virions) as the earlier models of Neumann and Dahari, but which has a significantly different structure to the steady state solutions. The typical bifurcation diagram for our model is shown in Figure 9 and consists of an uninfected steady state branch with a bifurcating branch of infected steady state solutions on which there is a single limit point. Allowing the bifurcation point to occur at positive or negative values of the treatment parameter means that the model can include spontaneous clearance, as well as relapse at the end of treatment. In the case S=0 (no generation of hepatocytes from stem cells), we showed that there is a pure infection branch of solutions, and an infected steady state branch was found analytically that connects the uninfected and pure infection branches. When S>0, the bifurcation between the pure infected and infected branches unfolds, generating a single valid branch of infected solutions on which there is a limit point (see Figure 7). We have been able to describe these solutions of the model using only the assumption (39) and the requirement that the quantity BP−D(B+C) is close to zero.

We have shown in Section 8 that our model is able to show the many profiles of viral load reported in the literature, and the model has been fitted to four datasets in Section 9. Moreover, based on the bifurcation diagram shown in Figure 9 for our model, we also made some predictions regarding treatment (see Section 7), which we summarise as follows:

- If the infection is caught and treated in the early stages, then our model predicts that a lower drug dose may be effective in eliminating the infection.
- If the viral load relapses on cessation of treatment, then continuing with a low level of drug treatment may keep the viral load low.
- The infected branch from the bifurcation on the uninfected branch to the limit point has a positive slope, and this suggests that the drug dose could be reduced as treatment progresses, which could save some of the costs of treatment and give a reduction in side effects for the patient.

The Neumann HCV model was obtained by modifying earlier models for HBV and HIV infections. It would now be interesting to see whether this process could be reversed by adapting this new model for HCV infection for other viral infections.

Another avenue of interest would be to fit this model to viral load data while a patient is on treatment in order to make patient-specific recommendations from the model regarding the future treatment plan. An important step in this process would be to determine the feasibility of estimating each of the parameters in the model, as has been done for other HCV models [28]. 

## Figures and Tables

**Table 1:** Parameter values for fitting the model to the data.

	PVR	Breakthrough	Null Response	Triphasic
*s* (day${}^{-1}$)	$1.1178\times 10^{-1}$	$1.5104\times 10^{-4}$	$4.6260\times 10^{-3}$	$3.1259\times 10^{-3}$
$r_{T}T_{\max}$ (IU/ml/day)	$1.0645\times 10^{4}$	$2.8556\times 10^{4}$	$1.2920\times 10^{6}$	$1.1149\times 10^{2}$
$r_{T}+d$ (day${}^{-1}$)	$1.9927\times 10^{-3}$	$2.9890\times 10^{-3}$	$3.8518\times 10^{-2}$	$1.7882\times 10^{-2}$
*R*	$3.0078\times 10^{1}$	$1.1686\times 10^{3}$	2.6011	$2.0350\times 10^{-1}$
*D*	$5.8954\times 10^{1}$	$5.7302\times 10^{2}$	1.1064	$1.0962\times 10^{1}$
$\beta^{*}$ (ml/IU/day)	$8.3376\times 10^{-9}$	$7.1149\times 10^{-9}$	$1.9493\times 10^{-7}$	$3.3281\times 10^{-8}$
$p^{*}$ (day${}^{-1}$)	$2.0396\times 10^{2}$	$9.4025\times 10^{1}$	$3.4868\times 10^{-2}$	$1.1646\times 10^{3}$
*c* (day${}^{-1}$)	$1.7908\times 10^{1}$	3.3659	$2.7784\times 10^{-4}$	1.4294
T(0) (IU/ml)	3.3246	$8.2935\times 10^{6}$	$1.7755\times 10^{4}$	1.9948
I(0) (IU/ml)	$4.1752\times 10^{5}$	$9.5880\times 10^{3}$	$1.4523\times 10^{6}$	$1.0355\times 10^{2}$
